# Social Reinforcement Delays in Free-Flying Honey Bees (*Apis mellifera* L.)

**DOI:** 10.1371/journal.pone.0046729

**Published:** 2012-10-04

**Authors:** David Philip Arthur Craig, James W. Grice, Chris A. Varnon, B. Gibson, Michel B. C. Sokolowski, Charles I. Abramson

**Affiliations:** 1 Department of Psychology, Oklahoma State University, Stillwater, Oklahoma, United States of America; 2 Department of Psychology, Université de Picardie – Jules Verne, Amiens, Picardy, France; Institut Pluridisciplinaire Hubert Curien, France

## Abstract

Free-flying honey bees (*Apis mellifera* L.) reactions were observed when presented with varying schedules of post-reinforcement delays of 0 s, 300 s, or 600 s. We measured inter-visit-interval, response length, inter-response-time, and response rate. Honey bees exposed to these post-reinforcement delay intervals exhibit one of several patterns compared to groups not encountering delays, and had longer inter-visit-intervals. We observed no group differences in inter-response time. Honey bees with higher response rates tended to not finish the experiment. The removal of the delay intervals increased response rates for those subjects that completed the trials.

## Introduction

Honey bees are a model organism for behavioral, neurobiological, and cognitive research [1]–[8]. Many phenomena such as spatial navigation, memory, learning, and social behaviors have been investigated in honey bees [9]–[11]. Further, honey bees provide a unique opportunity to study novel reinforcement contingencies, for they shuttle their reinforcing nectar to their hive and quickly return to a foraging location.

Here, we have developed a paradigm to study the effects of a post-reinforcement delay on honey bee (*Apis mellifera* L.) foraging behavior. We introduced a delay of reinforcement after the bee’s crop had been filled; responses made prior to the initiation of the delay intervals are hereafter referred to as crop-filling responses to remain consistent with Dyer, Gill, and Sharbowski [12]. Due to the cyclical rhythm of honey bee foraging behaviors, a post-reinforcement delay at a foraging location would in turn delay a variety of social behaviors in the hive such as unloading behaviors. Honey bees could easily experience long post-reinforcement delays under natural conditions. Instances such as severe winds, fires, or even becoming trapped within a flower could create a natural post-reinforcement delay; thus we have sought to assess the social contingencies that may be affected by such a delay. We believe honey bees are an ideal model to investigate post-reinforcement delays due to their rhythmic foraging behavior and unique social structure. Additionally, comparing different species’ cognitive and behavioral abilities is a legitimate investigation within itself.

Davis [13] is arguably the first to publish a finding focusing on post-reinforcement delays. His design utilized a T maze with a goal box that was modified to remove a rat 60 s after consuming a single reward 1/3 g pellet. The delay group did not differ from control subjects which were removed immediately after consuming the reinforcement [13]. Further work on post-reinforcement has not only been scarce, but has reached inconsistent conclusions.

Fehrer [14] tested rats in a U maze using water as the reward. Slower learning for the pre-reinforcement group was observed while the post-reinforcement group did not affect learning. Cogan’s [15] replication of Fehrer [14] confirms that there were no significant differences between post-reinforcement delay and no-delay groups during training. However, Cogan [15] found a decrease in resistance to extinction when the delay groups were compared to the control group; thus failing to replicate (and finding an opposite effect) [14]. Bowen [16] changed Fehrer’s experimental design by running rats through a T-maze instead of a U-Maze, and found rats who were immediately removed from the goal performed significantly better than rats treated with a 30 s post-reinforcement delay.

Additional rats with either a 0 s or 30 s post-reinforcement delay were allowed to run into a small or large goal box [17], [18]. Contrary to the observation made by Mikulka, Vogel, and Spear [17] regarding a larger goal box’s correlation with higher resistance to extinction, Williams [18] found no difference between confining and non-confining goal boxes. McCain and Bowen [19] attempted to determine how a small number of acquisition trials could produce a difference in groups of rats exposed to pre- or post-reinforcement. Both delay groups were more resistant to extinction compared to the immediately reinforced group, but no significant differences between the delay groups were observed (contrasting with Fehrer [14]). Rosen and Tessel [20] confirmed previous research [19] by showing no difference between post-reinforcement delay and no delay groups’ run times on a runway.

Capaldi, Godbout, and Ksir analyzed the effects of post-reinforcement delay intervals by dividing rats into three groups: continuous reinforcement, partial reinforcement, and no reward [21]. The researchers found a “marginal” level of significance between conditions [21]. Additionally, post-reinforcement delay rats had no observable patterning effects for start, run, and goal times compared to controls [22]. Indeed, a reverse patterning was observed for the delay group [22]. Moreover, Posey and Cogan [22] found that post-reinforcement delay subjects are less resistant to extinction; thus confirming previous research [15].

While, all previously reported post-reinforcement experiments have utilized rats as subjects, Rabinowitz and Paynter [23] analyzed differences in learning, relearning, and forgetting in 3rd grade students by using 6 s, 12 s, or 18 s of post-reinforcement delay. Children exposed to varying delays were faster at learning (for both genders), relearning (for only boys), and especially forgetting when presented with a distraction. On average, an increase of a post-reinforcement interval was associated with faster forgetting compared to pre-reinforcement delays [23].

We have developed a paradigm to study reinforcement delays utilizing uniquely species-specific behaviors in honey bees (*Apis mellifera* L.) by trapping our subjects after they had filled their crop in a computer controlled operant chamber. In addition to this post-reinforcement delay, our study differs from the post-reinforcement literature in multiple ways [13]–[23]. Most notably, our subjects were “wild” and could freely choose if they wanted to begin, continue, or stop working with our apparatus; indeed many subjects did not finish the trials. Second, counter to the majority of reinforcement procedures, our subjects were not food or water deprived. Third, our subjects were allowed to consume as much as they wanted, for there was no prescribed amount of reinforcement they could receive. Fourth, we offer the first data utilizing honey bees to investigate delays in social contingencies. Fifth, our paradigm to study post-reinforcement delays creates a subsequent delay of a social interaction: the unloading of reinforcement from our apparatus back at the hive by the forager. This unloading behavior is critical for a subject to be able to leave the hive and return to the apparatus, and often occurs between multiple receivers [24]. Additional social behaviors such as dancing and seeking a receiver for trophallaxis were likely also delayed by the paradigm [25]. Hence, a delay interval directly preceding a number of social behavior chains was created via our post-reinforcement delay experimental design.

## Results

We recorded four main dependent variables: inter-visit-intervals, defined as the interval between the last crop-filling response of a visit and the first response of the following visit; response length, defined as the length of time the subject makes a spatial response sufficient to block an infrared sensor; inter-response-time, defined as the interval between responses; and the response rate per visit. Response rate was calculated by only considering the crop-filling responses prior to the delay interval being initiated; hence the delay intervals did not artificially lower the reported response rates. We also recorded the temperature inside the apparatus every minute throughout each trial, as well as the date the subjects were tagged.

We utilized five groups and four conditions for each group with six visits per condition. We followed a pseudo-ABA design so as to be able to compare each subject with herself as we moved across the four conditions. The five groups are named after each of their varying four conditions (i.e. group 0-0-0-0 had no delay conditions, while the second condition for the 0-5-10-0 group had 5 minutes of delay and the third condition had 10 minutes of delay). Details of the apparatus and paradigm are provided in the Methods Section. Data were collected summer of 2011.

Every control 0-0-0-0 bee finished the 24 visits, but only four of the ten 0-10-10-0 bees, five of the ten 0-5-5-0 bees, six of the ten 0-5-10-0 bees, and five of the ten 0-10-5-0 bees finished the experiment by completing 24 visits. Every bee that did not return to the operant chamber was observed the following morning at a nearby 10% sucrose solution feeder; ruling out the possibilities of predation or death affecting our data.

Our experimental design makes data analysis by conventional methods impossible. First, our data do not meet the homogeneity assumptions made by traditional mean comparisons (Levene’s F = 13.193, p<.01). The control 0-0-0-0 group’s inter-visit-interval standard deviations are radically different from each of the experimental groups, for the delay intervals affected most (but not all) of the subjects; hence the greater variability for the experimental groups. Second, we utilized a repeated measures experimental design, yet many of our bees “dropped out” and thus a split-plot ANOVA would not be appropriate due to the “missing” data. Third, the difference between group baseline response rates indicate our sample and group assignment may not have been random, further compromising the validity of any p-value obtained from an ANOVA. Due to these complications, we eschewed traditional methods of data analysis and instead utilized a different method that is relatively free of assumptions and incorporates techniques for accommodating the “drop out” non-responses. We used Observation Oriented Modeling [26], [27]; a data analysis technique that permitted us to compare our observed results to expected patterns of outcomes and then to evaluate the differences with an accuracy index and a randomization or binomial test.

### Inter-visit-interval

**Figure 1:**
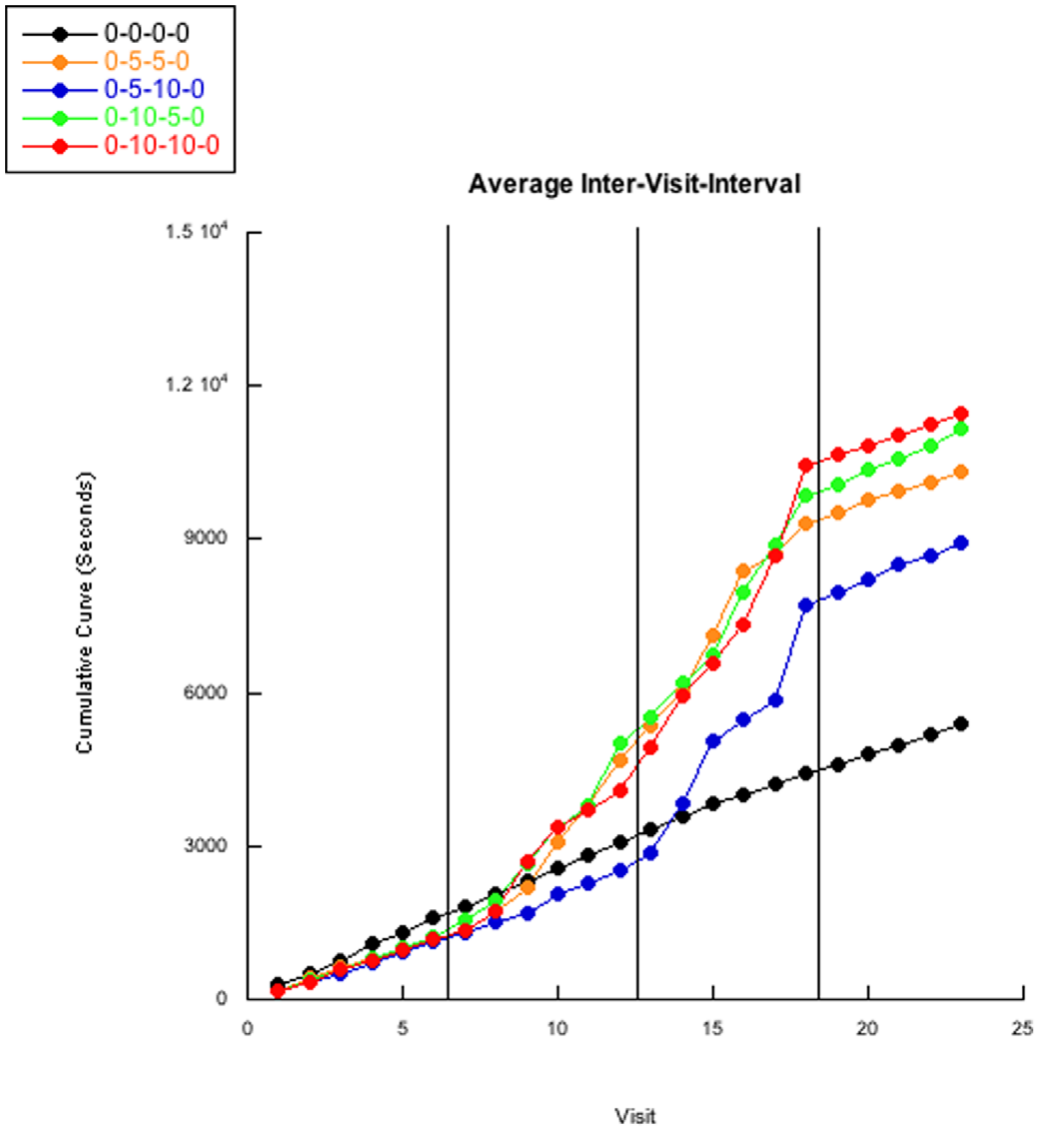
Averaged inter-visit-interval. The averaged cumulative curves of group inte*r-visit-interv*als are pr*esented. All g*roup’s baselines are very similar during *the first condition of the experiment. The aver*aged control 0-0-0-0 group maintains this increase for the remaining conditions and trials while the averaged 0-5-5-0, 0-10-10-0, and 0-10-5-0 experimental groups begin differing from the control group after about 3 visits of the second condition. The averaged 0-5-5-0, 0-10-10-0, and 0-10-5-0 experimental groups’ curves continue to increase (roughly resembling exponential curves when not presented as a cumulative curve) until the final condition change removing the delay intervals. Curiously, the averaged 0-5-10-0 group (which for the second condition is no different from the averaged 0-5-5-0 group) does not differ from the control group until the third condition (when the delay interval increases to 600 s), but then quickly increases similarly to the remaining experimental groups until the final conditional change removing the delay intervals. Most striking is the immediate near-return to baseline for all four experimental groups’ averages once there are no delay intervals. Table 2 displays average slope values for each condition and group for Figure 1. Individual bee’s cumulative curves can be observed in Figures S1,S2,S3,S4,S5.

Inter-visit-interval means and standard deviations reveal clear differences between the groups (Table 1). Cumulative curves of the inter-visit-intervals (Figure 1) were analyzed by regression analysis to determine slope differences within each condition and between groups (Table 2). Cumulative curves of individual bees were also analyzed via linear regressions (Figure S1, S2, S3, S4, S5). We observed three basic types of cumulative curve patterns for individual subjects: linear, exponential, and an “S” curve. “Drop out” bees never experienced the removal of the delay intervals, and thus never returned to baseline; thereby resembling an exponential “J” curve while experimental bees which did encounter a return to baseline resembled an “S” curve. Every control 0-0-0-0 bee followed a simple linear pattern. Nine of the ten 0-10-10-0 bees differed from every control subject; seven of the ten 0-5-5-0 bees differed from every control; eight of the ten 0-5-10-0 bees differed from every control; nine of the ten 0-10-5-0 bees differed from every control.

**Table 1:** Inter-Visit-Interval Descriptive Statistics for Each Group.

Group	Mean	Median	Mode	Range	SD
**0-0-0-0**	234 s	190 s	129 s	50 s–1377 s	183 s
**0-10-10-0**	481 s	237 s	3600 s	60 s–3600 s	721 s
**0-5-5-0**	441 s	197 s	3600 s	85 s–3600 s	667 s
**0-5-10-0**	358 s	202 s	200 s	50 s–3600 s	561 s
**0-10-5-0**	475 s	268.5 s	225 s	102 s–3600 s	650 s

**Table 2:** Slope of Cumulative Curves of Each Condition Per Group.

	1	2	3	4
**0-0-0-0**	264.11	249.8	220.23	192.22
**0-10-10-0**	198.47	586.34	1039.9	208.6
**0-5-5-0**	198.28	680.49	835.22	196.19
**0-5-10-0**	192.47	250.05	876.6	238.8
**0-10-5-0**	205.31	674.44	882.51	269.04

An ordinal pattern analysis was conducted for each group to assess if the inter-visit-intervals increased once the delays were initiated and then increased once the delays were removed. For bees in the experimental groups, we predicted the intervals would decrease during the baseline trials as the bees learned to work with the apparatus, and that the inter-visit-intervals would monotonically increase once the delays were initiated, and then would immediately decrease and nearly but not fully return to baseline after the delays were removed. This prediction was also confirmed by the slope differences obtained from our multiple regression reported (Table 2). For each bee, the analysis compares the differences between every possible pair of intervals to the hypothesized differences, and the percentage of responses that fit the predicted ordinal pattern is determined. Each interval is compared with every other interval for an individual bee (e.g. interval one vs. interval two, interval one vs. interval three…interval one vs. interval twenty-three, etc.); consequently, the number of responses that fit the ordinal pattern can range from 0 to _k_C_2_, where k equals the number of visits. For example, a bee completing 23 visits has 253 interval comparisons while a bee completing 18 visits has 153 comparisons to the expected ordinal pattern. The percentage of comparisons matching the expected patterns is computed for each bee, and a binomial probability is also computed.

Individual results of the ordinal analysis and contains proportions of combinations that matched the predicted pattern and the binomial p-values (Table 3). Consistent with expectations**,** seven of the ten control (0-0-0-0) bees did not fit the predicted experimental pattern. However, nine of the ten 0-10-10-0 bees, eight of the 0-5-5-0 bees, eight of the 0-5-10-0 bees, and all of the 0-10-5-0 bees followed the predicted pattern in improbable proximity compared to an arbitrarily selected cut-point of.05 for the binomial p-value.

**Table 3:** Inter-Visit-Interval Individual Results of Ordinal Analysis.

Group	Total	Bee1	Bee2	Bee3	Bee4	Bee5	Bee6	Bee7	Bee8	Bee9	Bee10
**0-0-0-0**	50%	**41%**	**55%**	69%	66%	**47%**	**42%**	55%	**36%**	**47%**	**45%**
		**p≤1.00**	**p≤.08**	p≤.00	p≤.00	**p≤.87**	**p≤.99**	p≤.05	**p≤1.00**	**p≤.84**	**p≤.93**
**0-10-10-0**	74%	**55%**	83%	84%	66%	75%	69%	86%	71%	79%	80%
		**p≤.08**	p**≤**.00	p≤.00	p≤.00	p≤.00	p≤.01	p≤.00	p≤.00	p≤.00	p≤.00
**0-5-5-0**	61%	80%	65%	65%	**34%**	69%	**33%**	84%	83%	78%	74%
		p≤.00	p≤.00	p≤.00	**p≤1.00**	p≤.01	**p≤1.00**	p≤.00	p≤.00	p≤.00	p≤.00
**0-5-10-0**	62%	82%	62%	**39%**	62%	56%	67%	64%	67%	**51%**	86%
		p≤.00	p≤.02	**p≤1.00**	p≤.02	p≤.03	p≤.00	p≤.00	p≤.00	**p≤.35**	p≤.00
**0-10-5-0**	74%	82%	78%	77%	78%	63%	68%	83%	80%	81%	61%
		p≤.00	p≤.00	p≤.00	p≤.00	p<.00	p≤.00	p≤.00	p≤.00	p≤.00	p≤.05

### Weather Variability

**Figure 2:**
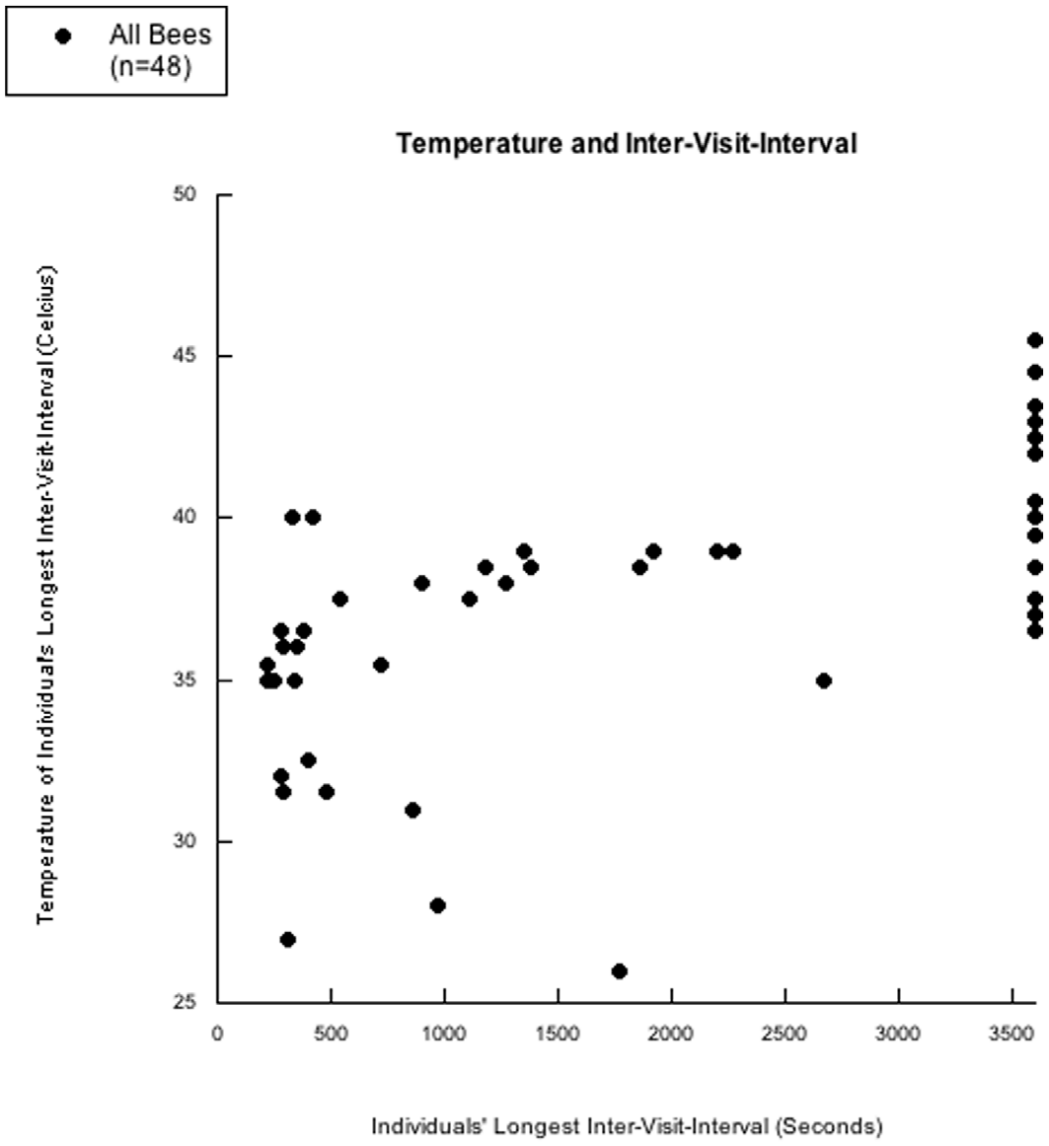
Temperature and inter-visit-interval. A scatter-plot of every individual bee’s longest inter-visit-interval and the temperature inside the operant chamber of the apparatus during an individual bee’s longest inter-visit-interval is presented. Two subjects did not have any temperature data. No bees above 40 degrees finished the experiment. However, long inter-visit-intervals can still be observed for bees with comparatively lower temperatures; indeed the subject with the lowest temperature had a higher inter-visit-interval than more than half of the bees.

Heinrich [28] reports honey bees are capable of foraging at temperatures as high as 46C without over-heating. As temperature has been shown to affect honey bee behavior [28], we recorded temperature at single-minute intervals during the experiment to assess temperature effects on our various DVs. Our data logger did not record temperature data for two out of 50 subjects. Of these 48 subjects with temperature data, 18 subjects did not complete the 24 trials. The maximum temperature of the 18 subjects that did not finish the experiment ranged from 36.5C to 45.5C while the temperature range for the 30 subjects that did finish the experiment varied from 26C to 40C. Figure 2 contains a scatter plot of the temperature associated with each bee’s longest inter-visit interval; Figures S6,S7,S8,S9,S10 display scatter plots for individual bees.

An ordinal analysis comparing temperature for bees that finished the trials versus those that did not return to the apparatus within 3,600 s (one hour) was performed. The analysis was run under the prediction that drop-out bees would have higher temperatures compared to the temperature paired with the longest inter-visit-interval for each bee that did finish the trials. Drop-out bees had higher maximum temperatures for 90% of the matches in the ordinal analysis (c<.01). We then separated by group to determine if drop-out bees had higher maximum temperatures within groups in addition to between groups. As the control 0-0-0-0 group had no “drop-outs,” we only analyzed the four experimental groups. Within every experimental group, every “drop-out” bee had a higher maximum temperature than the temperature paired with the longest inter-visit-interval for bees that finished the experiment.

### Response Length

An ordinal analysis of response length was conducted to test if response length was smaller after the subject’s crop had filled and the delay interval had been initiated. Every response prior to the delay interval being initiated was compared to every response after the delay interval was initiated, and a randomization test was performed to determine if the differences indicated consistently shorter durations. The 0-0-0-0 control group bees were not considered for this analysis as no adjunctive responses were made in the 0-0-0-0 control group. Individual and group percentages are displayed in Table 4, and most were over 90%; indicating a high degree of pattern matching. All chance values from the randomization tests were less than.01.

**Table 4:** Percentage of Adjunctive Responses Which Are Smaller Than Crop-Filling Responses.

Group	Total	Bee1	Bee2	Bee3	Bee4	Bee5	Bee6	Bee7	Bee8	Bee9	Bee10
**0-10-10-0**	91%	79%	94%	83%	93%	93%	96%	97%	*	84%	98%
**0-5-5-0**	92%	65%	91%	98%	91%	*	99%	100%	74%	*	96%
**0-5-10-0**	89%	*	*	98%	86%	89%	98%	85%	99%	95%	62%
**0-10-5-0**	92%	*	97%	97%	97%	89%	98%	100%	95%	96%	91%

* subjects did not make additional responses.

We also used an ordinal analysis to determine if the first response of a visit just after a visit containing the seemingly adjunctive responses was longer than trials not coming after an additional or adjunctive response. We did not analyze bees that did not make adjunctive responses, nor did we analyze bees that did not return after a single adjunctive response as these subjects had no data to make an ordinal comparison. Table 5 contains group and individual percentages of the first response following a series of adjunctive responses being larger than first responses of a visit not following an adjunctive response (all c values <.01).

**Table 5:** Percentage of Larger First Responses Following an Adjunctive Response.

Group	Total	Bee1	Bee2	Bee3	Bee4	Bee5	Bee6	Bee7	Bee8	Bee9	Bee10
**0-10-10-0**	92%	98%	95%	89%	77%	99%	*	97%	*	64%	100%
**0-5-5-0**	83%	100%	67%	84%	86%	*	100%	100%	80%	*	81%
**0-5-10-0**	83%	*	*	91%	8%	98%	82%	41%	86%	98%	64%
**0-10-5-0**	83%	*	78%	82%	95%	76%	87%	100%	100%	66%	44%

* subjects either did not make an additional response or did not return after an additional response.

### Inter-response-time

**Figure 3:**
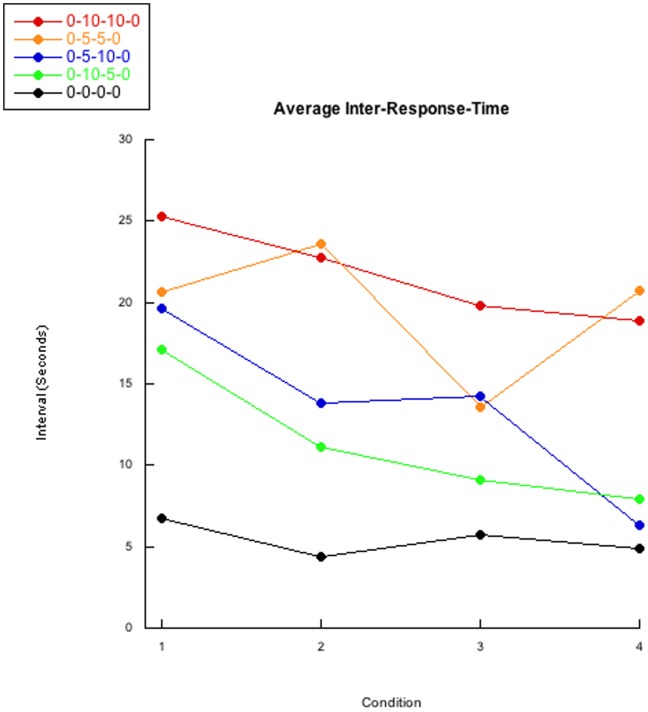
Average inter-response-time. The averaged group inter-response-times are presented. Save the average of the 0-5-5-0 experimental group, an overall downward trend can be observed. Curiously, the average of the control 0-0-0-0 group’s baseline IRT is much lower than the four experimental group’s.

A graphical representation of the collected IRT data did not show any apparent differences between groups; though a slight decrease in IRT group averages per condition could be interpreted (Figure 3). Individual bee’s IRTs are displayed in Figures S11,S12,S13,S14,S15. To test if there were indeed no differences between groups, we conducted an ordinal analysis in Observational Oriented Modeling. We predicted a decrease between conditions, but not within conditions, and no difference between groups. An analysis similar to our investigation of the inter-visit-intervals found highly similar results between groups, but not within groups as great variability in IRTs within groups was observed.

Table 6 contains individual binomial p-values and percentages of responses following the predicted pattern of the ordinal analysis. Overall, the similar group pattern matched percentages led us to disregard group differences in IRT. After adjusting the predicted patterns within condition, we determined these lower percentages of matches were due to variability within condition and within bees; however, the similarities between groups were still observed.

**Table 6:** IRT Pattern Matching of Individual Bees.

Group	Total	Bee1	Bee2	Bee3	Bee4	Bee5	Bee6	Bee7	Bee8	Bee9	Bee10
**0-0-0-0**	47%	**56%**	**57%**	42%	**55%**	27%	44%	46%	54%	54%	37%
		**p≤.02**	**p≤.02**	p≤1.00	**p≤.05**	p≤1.00	p≤.98	p≤.90	p≤.10	p≤.10	p≤1.00
**0-10-10-0**	44%	42%	23%	38%	47%	**59%**	28%	50%	44%	50%	27%
		p≤1.00	p≤1.00	p≤1.00	p≤.82	**p≤.00**	p≤1.00	p≤.56	p≤.89	p≤.58	p≤1.00
**0-5-5-5**	33%	31%	22%	31%	36%	0%	48%	38%	50%	16%	29%
		p≤1.00	p≤1.00	p≤1.00	p≤1.00	p≤1.00	p≤.78	p≤1.00	p≤.54	p≤1.00	p≤1.00
**0-5-10-0**	42%	41%	33%	25%	42%	41%	50%	21%	34%	**65%**	**65%**
		p≤.99	p≤1.00	p≤1.00	p≤.95	p≤.99	p≤.58	p≤1.00	p≤1.00	**p≤.00**	**p≤.00**
**0-10-5-0**	44%	22%	50%	20%	50%	**65%**	59%	39%	27%	26%	50%
		p≤1.00	p≤.48	p≤1.00	p≤.57	**p<.00**	p≤.00	p≤.93	p≤1.00	p≤1.00	p≤.55

### Response Rate

**Figure 4:**
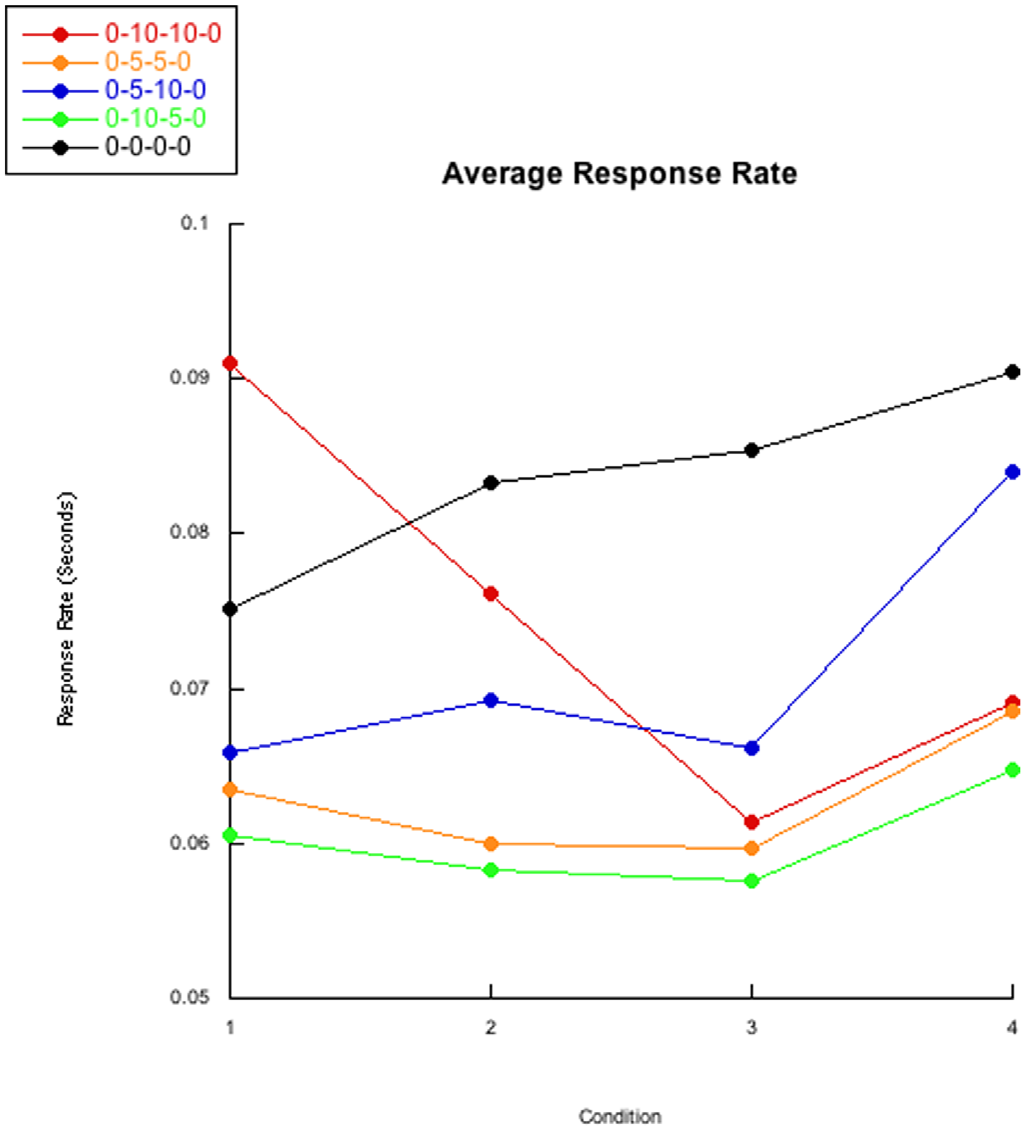
Average response rate. The condition averages of group response rates are presented. The average of the control 0-0-0-0 group displays a subtle rise across conditions while the experimental groups do not display this increase during the second and third conditions. The removal of the delay intervals for the experimental groups induces a rise in response rate. The average of the baseline for the 0-10-10-0 group differs from the other groups due to one powerful “outlier” which dropped out mid-way through the second condition (visible in Figure S18).

We predicted an increase within and between conditions, for if subjects acclimated to the apparatus, response rates could be expected to increase. This prediction is confirmed by a post hoc analysis of average response rates based on the control 0-0-0-0 group’s increase in response rates; visible in Figure 4. Figures S16,S17,S18,S19,S20 contains individual bee’s response rates.

An ordinal analysis similar to our investigation of the inter-visit-intervals and IRT found highly similar results between groups, but not within groups. Variability in response rates within groups was observed; Table 7 contains individual binomial p-values and percentages of responses following the predicted pattern of the ordinal analysis. Overall, experimental conditions all differed from the control group, and while differences between experimental groups were observed, these differences were not substantively important.

**Table 7:** Response Rate Pattern Matching of Individual Bees.

Group	Total	Bee1	Bee2	Bee3	Bee4	Bee5	Bee6	Bee7	Bee8	Bee9	Bee10
**0-0-0-0**	66%	**69%**	**69%**	**64%**	**55%**	**71%**	**60%**	**58%**	**72%**	**72%**	**67%**
		**p≤.00**	**p≤.00**	**p≤.00**	**p≤.05**	**p≤.00**	**p≤.00**	**p≤.01**	**p≤.00**	**p≤.00**	**p≤.00**
**0-10-10-0**	49%	45%	29%	34%	43%	**71%**	**67%**	**58%**	54%	60%	44%
		p≤.95	p≤.100	p≤1.00	p≤.92	**p≤.00**	**p≤.03**	**p≤.03**	p≤.29	p≤.03	p≤.81
**0-5-5-5**	55%	**56%**	49%	**66%**	52%	31%	**72%**	33%	**60%**	24%	41%
		**p≤.02**	p≤.62	**p≤.00**	p≤.26	p≤1.00	**p≤.00**	p≤1.00	**p≤.02**	p≤1.00	p≤.95
**0-5-10-0**	57%	**71%**	**55%**	**63%**	43%	41%	47%	43%	52%	**67%**	**68%**
		**p≤.00**	**p≤.05**	**p≤.00**	p≤.93	p≤1.00	p≤.78	p≤.94	p≤.29	**p≤.00**	**p**≤**.00**
**0-10-5-0**	59%	39%	**58%**	**55%**	45%	**83%**	**78%**	58%	42%	26%	**79%**
		p≤.99	**p≤.01**	**p≤.05**	p≤.95	**p<.00**	**p≤.00**	p≤.20	p≤.91	p≤1.00	**p≤.00**

The averages plotted in Figure 4 suggest the 0-10-10-0 group has a far different baseline compared to the other groups. Consequently, we investigated if there were differences in response rates within groups when comparing bees that finished the experiment with bees that did not finish the experiment. Thus, we predicted bees that dropped out had a higher response-rate per visit and tested this prediction with an ordinal analysis similar to how we previously analyzed response length. This analysis compared each response rate from the bees that did not finish the experiment with the response rates from the bees that did finish the 24 trials. As every bee in the control 0-0-0-0 group finished all 24 trials, we did not analyze the control group’s response rates. Table 8 displays percentages of drop-out bee’s response rates that are larger than response rates from bees that finished the experiment. While a difference between drop-out bees and bees that finished the experiment can be observed when comparing all 24 responses, we do not choose to interpret a predictive quality of response rates as gauged from baseline trials. Phrased differently, we do not believe larger baseline response rates appropriately predict if a subject will not complete the 24 trials.

**Table 8:** Percentage of Drop-Out Bees with Higher Response Rates.

Group	Baseline	All Visits
**0-10-10-0**	60%	60%
	c≤.00	c≤.00
**0-5-5-0**	37%	45%
	c≤.10	c≤.00
**0-5-10-0**	14%	81%
	c≤1.00	c≤.00
**0-10-5-0**	60%	52%
	c≤.00	c≤.00

### Shaping Latency

We documented the latency between shaping/tagging a bee and initiating the experiment for each bee. On occasion, we shaped and ran a subject on the same day; however, we also frequently tagged bees (up to nine) days prior to running a subject. A regression of this latency on to whether the subject finished 24 trials found a R2 value of.30. An ordinal analysis in Observational Oriented Modeling found negligible latency effects between bees that finished and did not finish the experiment; 30% prediction match, c<.17. Latency between tagging and data collection was regressed on to the number of additional responses made after the delay intervals were initiated found a R2 value of.16.

## Discussion of Dependent Variables

### Temperature and Inter-visit-interval

We were concerned about the covariance between temperature and inter-visit-interval. Prior to beginning any data collection, we made a schedule for 50 bees and counter-balanced subject order in an attempt to control for weather variability. Data collection would start for each bee usually around 10∶00, but control 0-0-0-0 bees would often finish before 12∶00 while experimental groups often finished around 16∶00. Thus, temperatures were far higher for the experimental bees during the end of the experiment; thus exacerbating the temperature difference between groups. Regardless, Figure 2 clearly shows bees did not finish the experiment if the temperature exceeded 40.5C. We believe investigating this temperature effect is crucial, but at this time we are unsure of how temperature in combination with our delay intervals may function to dissuade subjects from returning to the operant chamber. Most notably, while temperature certainly affected and accounts for at least some variability in the inter-visit-intervals for groups and individuals, the very clear decrease of the inter-visit-interval cumulative curve’s beta-weights (for individuals and groups) once the delay intervals were removed demonstrates the impact of the delay, and functions to show how the delay impact is more salient than that of temperature. Further demonstrating that temperature did not contaminate our data would be the control groups’ decrease in inter-visit-intervals across individual bee’s trials, for even though the temperature increased across conditions, a decrease was observed in inter-visit-interval for the 0-0-0-0 control group (as seen in Table 2).

Regardless, the effect of temperature on the experimental bees is undeniable; thus we propose temperature moderates the inter-visit-intervals. The relatively low percentage of our observations matching our proposed model may seem disconcerting; however, our pattern yielded far more correct responses for the first three conditions for each bee, yet the return to baseline drastically reduced our percentage match as many of the responses fully returned to, or out-performed, baseline (differing from our pattern analysis). Also, our criteria are far more strict than any traditional statistical analysis; any data deemed “significant” in Observational Oriented Modeling will also be significant in any traditional statistical test. Most importantly, in addition to predicting a difference between groups, we also predicted the direction and the order of these differences. No other analytical method we know of is capable of testing such a complicated ordinal prediction while not making numerous assumptions. Regardless, our prediction was corect (based on an arbitrary value of p<.05) for 35 out of 40 experimental group bees. Thus, we feel confident stating that the delays increase inter-visit-intervals. Subjects first encountering a 600 s delay performed more poorly than subjects first encountering a 300 s delay. We did not show a difference in inter-visit-intervals between the 0-5-5-0 and 0-5-10-0 groups, nor did we show a difference in inter-visit-intervals between the 0-10-10-0 and 0-10-5-0 groups.

### Response Length

Our analysis of response length yielded two major findings. First, a decrease in response length was clearly observed for nearly every response made after the delay had been initiated. Only two out of 34 bees did not match this prediction based on an arbitrary value of more than 66% matching. After comparing the length of the adjunctive responses to the normal crop-filling responses, we do not believe the subjects were receiving reinforcement during their post-delay responses, for the response lengths are usually impossibly small for a subject to make the response and consume the reinforcement. Indeed, we observed sugar water dripping within the apparatus following a string of these adjunctive responses; validating the possibility of this hypothesis. Our second analysis of response length, which found that the first response of a new visit following a string of adjunctive responses was larger than responses not following an adjunctive response, is related to our first response length analysis. We believe the subjects were not consuming some or most of the reinforcement during the responses during the delay interval, and thus when the subjects returned to the operant chamber for the next trial we observed longer response lengths for the first response as the subjects consume the previous visit’s left-over reinforcement; hence our utilization of the term “adjunctive response.” Thus, a very clear difference between the normal crop-filling responses prior to the initiation of the post-reinforcement delay and the responses during the delay can be observed and inferred from two response length analyses.

### Inter-Response-Time

The benefit of our focus on individual observations instead of focusing on aggregate data is best exemplified when interpreting IRT data. When considering Figure 3, a slight monotonic decrease in group averages across conditions can clearly be observed for every group other than the 0-5-5-0 experimental condition. However, when considering individual bees, only 5 of 50 bees (10%) followed this aggregate-based prediction as determined by our binomial p-value <.05. Only one 0-0-0-0 control bee followed the predicted pattern, indicating the experimental groups’ 36 bees which departed from our expected pattern did not do so due to the post-reinforcement delays. However, the similarity between the groups’ percent matching dissuades us from claiming IRT is affected by our post-reinforcement delays. Consistent with our findings, pigeons with long delays have previously been observed to have a monotonically inverse relationship between pre-reinforcement delay length and IRT (e.g. [29]–[31]).

### Response Rate

Our initial analysis of response rate tested if, as observed in the control 0-0-0-0 group, experimental groups’ response rates monotonically increased across visits. Every control 0-0-0-0 bee was found to follow the predicted response rate increase, while only 18/40 experimental bees followed the predicted response rate increase. Experimental groups matched the predicted pattern approximately equally and clearly differed from the control 0-0-0-0 group. When considering Table 8, a very clear dichotomy can be observed in the bi-nominal p-values for individuals in the experimental groups. However, the 0-10-10-0 group differed furthest from the control 0-0-0-0 predicted pattern, for this experimental group had a powerful “outlier” affecting the mean of the group’s response rate (as seen in the baseline of Figure 4).

We decided to investigate these differences in baseline and found, for the 0-10-10-0 and 0-10-5-0 groups, that bees with higher response rates tend to drop out more than bees with smaller response rates. Interestingly, these differences in baseline performance for future drop-outs were not observed for the 0-5-5-0 or 0-5-10-0 groups. Because of this inconsistency, we do not believe higher baseline response rates can predict longer inter-visit-intervals when the subjects encounter the delays. However, we decided to compare every visit’s response rate for bees that dropped out of the experiment with bees that completed all 24 trials for each group, and found an apparent difference between bees that dropped out and completed the trials for all experimental groups’ response rates. In short, bees with higher response rates were more likely to “drop-out” when exposed to the delay intervals. While “patience” could play a factor, we believe bees with higher response rates would have higher metabolisms and thus require more reinforcement more quickly. Thus, not receiving this required sustenance may disincline bees with higher response rates from continuing to work in a delayed contingency.

The most obvious finding regarding response rate is the immediate response rate increase once the delays have been removed for group (Figure 4) and individual bees. Clearly, our delays were affecting response rate as the delay removal created a sudden increase in response rate across all groups. Thus, we posit that extensive post-reinforcement delays impair response rate increases traditionally seen in non-delayed subjects, or at the very least serve to increase response rates once the delay has been removed.

### Shaping Latency

The latency between tagging and beginning data collection for individual bees clearly had a negligible impact on the reported effect. Thus, we believe the interval between shaping and beginning data collection have little impact on our positive findings.

## General Discussion

There are five major differences between our experiment and previous post-reinforcement delay research [13]–[23]. First, we focused on acquisition trials. Second, we used a small number of acquisition trials (six) before presenting our subjects with reinforcement delays. A third crucial difference is the species under investigation, for all but one study [23] we found on post-reinforcement delay literature used rats [13]–[22]; we used honey bees: *Apis mellifera* L. A fourth major difference between the present study and the traditional post-reinforcement paradigms is our utilized delay times. The literature typically reports either 20 s or 30 s of post-reinforcement delay with the exception of a 60 s interval by Davis [13]. We used 300 s and 600 s intervals (a 100× and 200× increases compared to the literature). Our decision to use these large delays follows the tendency of typical extinction trials to last 10 minutes [32], for we believe any actual behavioral effects caused by post-reinforcement delays would be best observed this way. Finally, our subjects were still able to, and at times did, feed after the post-reinforcement delay had been initiated; no other study allows reinforcement to be provided during the delays.

An alternative explanation of our data is the concept of motivation. There are a couple of paradigms which are closely related to the present paradigm. These paradigms sought to consider “motivation” when constructing bee foraging models [33]. Most similar is Dyer, Gill, and Sharbowski [12], which delayed honey bees with and without access to sucrose for either 0 seconds, 30 minutes, or 3 hours, and released these bees either from the original feeding location or a novel location. They report the majority of subjects were observed flying directly home from the release site, but found subjects that were delayed 3 hours without access to food did not fly to the hive, but instead flew in the opposite direction of the hive; hence they flew as if they were leaving the hive and attempting to return to the foraging location. We observed all of our honey bees fly directly to the hive for every trial; consistent with previous findings [12]. Moffatt [34] has demonstrated the reinforcement rate as opposed to the crop-weight affects honey bee metabolic rates; hence our paradigm could be argued to have lowered the “motivation” of subjects due to the decrease in response rate per visit (when accounting for the delay intervals); possibly affecting the inter-visit-intervals. Additionally, Chen, Hung, and Yang [35] exposed honey bees to stressful environments after the subjects had fed at a feeding location. Experimental bees had up to 10× longer inter-visit-intervals compared to control groups. Changes in the experimental bees’ biogenic amines indicated the subjects were likely stressed by the post-reinforcement environment resulting in longer inter-visit-intervals. Indeed, one could argue, based on the reported honey bee literature, the present study’s delay intervals affected honey bee “motivation.” However, our delay intervals of 5 or 10 minutes would assumingly not be as “unmotivating” as a 30 minute or 3 hour delay. However, over time, these smaller 5 or 10 minute delays still could be argued to have “unmotivated” the subjects, resulting in longer inter-visit-intervals.

We are disinclined to offer “motivation” as a primary mechanism contributing to the observed effects. The subjects were rewarded with 50% sucrose, an extremely potent concentration compared to what is readily available in the natural environment. Also, the distance subjects had to travel from the hive to the apparatus is very short (approximately 20 m), and all of the rewards occur in a single location. Typically to fill its crop, a bee may travel an average of two or three kilometers. Subjects also have to visit many flowers containing comparatively low sugar molarity rarely over 25% sucrose; thus it is highly unlikely that the subjects would give-up such a rich, plentiful, and easily obtainable food source, and we are hesitant to infer any “motivational” effect [36]–[38]. Additionally, over a 25 year period, Wells [39]–[42] has reported results using blue and yellow artificial flower patches which elicit high flower fidelity for flower color by visiting bees. Some bees visit only blue and some visiting only yellow flowers regardless of the rewards. Moreover, if bees receive 2 M sucrose on one flower color and 1 M (or less) on the other flower color the return rates of the bees visiting the same flower patch at the same time are equivalent. “Motivation” has often been a label of calories per unit time, and if this is indeed driving our subject’s behavior, then having a longer return time would have the opposite effect of an efficient or “motivated” organism because the subjects would be further reducing the calories per unit time by having longer inter-visit-intervals. Due to these factors, we instead turn to the possibility that our post-reinforcement delays also served to delay social contingencies in the hive.

“Social reinforcement” in the non-human species literature has largely only been investigated in *Betta splendens* [43], [44]. These investigations exposed Siamese fighting fish to a mirror or conspecific as a reward; indeed these are the only operational definitions of “social reinforcement” in non-human literature. These definitions eventually evolved into investigations into aggression and focused on the negative reinforcement incurred via the release of this aggression [45]. Bronstein [45] argues against the development of global definitions of social reinforcement due to the sheer diversity of the possible behaviors requiring such a label. Regardless, based on a definition of social behaviors, we generally define “social reinforcement” as a form of reinforcement being delivered to a subject via an interaction or observation of an interaction with another living entity [45]. Specifically for honey bees and our paradigm, social positive reinforcement is provided to the foraging subjects by the receiving hive-mate during the unloading process following the forager’s return from the operant chamber. We decided to focus on the unloading behavior in this definition because foragers are required to unload in order to leave the hive [46]–[50]. Other social behaviors (e.g. trophallaxis, dancing, begging, and recruitment) need not necessarily be performed in order for the forager to leave the hive and return to the foraging location. This proposed social reinforcement is not presently immediately observable, and many possibilities (e.g. food, pheromone, and associations) need to be examined before further specifying the mechanism of reinforcement.

In a similar paradigm to the present study, Wainselboim, Roces, and Farina [51], investigated how the flow of trophallaxis may be affected by perceived value of a food source. Most importantly for our purposes, the researchers predicted and found that longer visit lengths resulted in slower rates of trophallaxis [51]. Farina [25] also reports forager begging after unloading just prior to leaving the hive is less frequent at higher rates of nectar flow. We were unable to observe if our subjects were engaging in trophallaxis or begging; though a slower rate of trophallaxis and an increase in begging behavior may account in part for the longer inter-visit-intervals and also communicate the temporal value of the operant chamber with delays as less reinforcing than the operant chamber without the delays. Additionally, flight times between conditions did not meaningfully alter for the delayed subjects as each subject was observed directly flying between the hive and operant chamber, hence the increase in inter-visit-interval occurs in the hive.

We were unable to observe our subjects’ behavior once they returned to and entered the hive, and thus trophallaxis and begging behaviors were not directly observed. However, it is generally accepted that foraging honey bees engage in unloading behavior prior to leaving the hive; thus unloading need not be observed [22], [46]–[50]. Thus, we sought to consider the impacts of the social interactions of our subjects in the hive on our results. Our design utilizes the rhythmically cyclical nature of honey bee foraging, hence a post-reinforcement delay will delay the social interactions back at the hive thus creating a delay of various forms of social reinforcement incurred from these interactions. Via this interaction, our post-reinforcement delay paradigm could also be an investigation in to a delay of social reinforcement incurred via interactions with hive mates.

Future studies should first attempt to replicate our observations. Determining honey bee sensitivity and reactions to these types of delays is crucial before conducting further studies into their sensitivity of various operant paradigms. We also recommend utilizing a choice paradigm to investigate if honey bees favor smaller delay intervals. Using an observational hive for future studies is also highly recommended so as to be able to observe the subjects unloading behavior. Diversifying the honey bee species used to study post or social reinforcement delays is also critical, for sub-species differences in foraging behaviors have been observed [52]. We also recommend comparing our findings with other hive insects that engage in similar unloading behaviors such as ants, wasps, or termites. The applicability of this paradigm on various human behaviors, such as incarceration, seems appropriate; though more comparative research should precede this speculation.

## Materials and Methods

### The bees

Subjects were *Apis mellifera* L. (n = 50) from the Oklahoma State University Comparative Psychology and Behavioral Biology Laboratory apiary. Bees from two different hives, which were roughly a half meter from each other, were trained to visit an artificial feeder. As Oklahoma State University does not require an ethics institutional review for non-threatened invertebrates, no specific permits were required. All subjects were experimentally naive prior to shaping. Subjects were randomly assigned to one of five groups receiving varying amounts of post-reinforcement delays. We decided to have 10 subjects per group to remain consistent with previous free-flying operant studies from our laboratory [7], [53]. Nine previous studies [13]–[22] investigating post-reinforcement delays utilized fewer subjects than we did. Performing a power analysis was not an option to determine an appropriate N as we sought to analyze individual subject data instead of averaged group data. We followed a pseudo-ABA design so as to be able to compare each subject with itself as we moved across conditions. We followed this type of ABA design in a previous study from our laboratory assessing how ethanol affects honey bee foraging behavior [7]. This study saw numerous differences between individual subjects; thus comparing each subject to itself will yield more reliable data than simply averaging group data [7].

A baseline of a 0 s delay was administered for the first and last six visits for each bee for every group while the middle 12 visits varied per group depending on the amount of post-reinforcement (expressed in minutes). The groups were as follows: 0-0-0-0, 0-5-5-0, 0-10-10-0, 0-5-10-0, and 0-10-5-0 with each number representing the delay interval encountered by the group for each phase of six visits. Two subjects from different groups were run concurrently in two separate yet attached operant chambers so as to control for weather conditions and other unforeseen biases associated with one operant chambers being slightly closer to the hive than the other. Subjects were trapped and terminated as soon as the experiment was concluded so as to control for recruiting and other unforeseen confounds such as pheromone release.

### Apparatus

We concurrently utilized two automated computer-controlled operant chambers providing 50% sucrose solution which was located approximately 3 meters from a feeding station containing 10% sucrose solution. Data was recorded automatically via a computer program. Subject responses were made when the subject enters a hole in the operant chamber with an infrared sensor which, when triggered, released a prescribed 5µl of sugar water directly in front of the subject’s head. Due to the infrared sensor’s sensitivity and disruption of functionality brought about by direct exposure to sunlight, we utilized a tarp which was placed in a consistent location every morning before beginning the experiment. A full explanation of the apparatus and calibration data can be found in [53]; though we slightly modified this design to be able to concurrently collect data from two subjects via two attached, partitioned operant chambers.

We also used a data-logger which was never left in direct sunlight, and was placed in the operant chamber to better measure weather variables (specifically temperature) for the subjects.

### Shaping

Subjects were randomly collected from the nearby feeder station equipped with 10% sucrose solution and shaped to use the apparatus described above. The feeding station was always full during the experiment to control for recruiting confounds. Training took no more than 10 visits. We typically focused on shaping two bees by first placing drops of sugar water near the response hole and then in the response hole. Sometimes we were obliged to hand-shape a subject into the response hole while other subjects were able to auto-shape themselves. After these bees had been trained to make the response, the newly trained bees were able to recruit additional potential subjects; exponentially increasing the number of trained bees in a matter of minutes.

Once the bee-in-training consistently returned to the operant chamber, we tagged her so as to be sure we were working with the intended subject. We used a Queen Marking Tube (QMT1) to securely keep the subject immobilized and attached a colored and numbered bee tag by using a safe, non-toxic adhesive. These materials were purchased from Betterbee® (Greenwich, NY). We attempted to minimize the time the subject was in the Queen Marking Tube to reduce what we assume to be a stress inducing aspect of our procedure. Once the subject was tagged, we provided her with three drops of 50% sugar water to combat this assumed tagging punishment before allowing her to return to the hive. We normally tagged numerous bees in one day and returned over the following days to administer our experiment until we needed to spend another day shaping and tagging our future subjects. Frequently, one of the bees would finish her experimental trials before the other concurrently run bee, in which case we shaped and trained more bees whilst finishing the experiment with the remaining bee. We recorded the date that each bee was tagged.

### Post-reinforcement Delay Test

We worked with the first two bees that came to the apparatus each morning after we had set up the apparatus. Two bees were run each day for 24 visits/trials apiece. Six baseline trials of a 0 s post-reinforcement delay was administered; allowing each bee to serve as her own control. During this time, subjects were allowed to freely exit the operant chamber once her crop filled. Beginning with the 7th and ending with the 12th visit, a bee was confined in the box for either 0, 5, or 10 minutes depending on her group assignment. Conditions and delay intervals changed again at the 13th visit and were held consistent until the 18th visit. Beginning with the 19th visit, we allowed the subjects to once again freely exit the operant chamber; returning to a 0 post-reinforcement delay.

We started the delay interval after the subject had finished feeding and attempted to fly out of the blocked/closed operant chamber. During the delay periods, subjects were free to continue making responses, but these responses did not reset the delay interval. Sometimes a subject would not leave the box after the post-reinforcement delay and in these cases we forced her to exit by gently removing her from the operant chamber with a small fish aquarium net. A session was terminated if the subject failed to return to the apparatus after one hour, or if we saw her return to the 10–12% sucrose solution feeder which we monitored through-out the experiment. In the event of a terminated session we checked the nearby bee feeder the following day to document the possibility of predation or death as the reason for their lack of return to the apparatus.

### Statistical Analyses

We used Observation Oriented Modeling [26], [27] which is a data analysis technique that permitted us to compare our observed results to expected patterns of outcomes and then to evaluate the differences with an accuracy index and a randomization or binomial test.

## Supporting Information

Figure S1 **0-0-0-0 Individual Inter-Visit-Interval.** All ten control 0-0-0-0 individual cumulative curves are presented. Bee 10 is the only subject not following pattern mode for the control 0-0-0-0 group; indeed a learning curve could be interpreted. Bee 10 begins resembling the other 9 control 0-0-0-0 bees by visit 15.
(TIF)

Figure S2 **0-5-5-0 Individual Inter-Visit-Interval.** All ten experimental 0-5-5-0 individual cumulative curves are presented. All ten experimental 0-5-5-0 bees’ baselines are very similar. Five bees finished all 24 trials with five dropping out. Bees 5 and 9 dropped out at visit 9; Bee 10 at visit 12; and Bee 7 and 8 at visit 16. Bee 5 is the only bee that did not return to the nearby bee-feeder instead of the apparatus; hence a jump of 3600 s is not observed for this cumulative curve. Bees 3, 4, and 6 were not affected by the delay intervals. The remaining two bees that were affected by the delay intervals and did finish the 24 trials both saw an immediate near-return to baseline once the delay intervals were removed. Bees 1, 8, 9, and 10 began to depart from baseline performance as early as visit 8. Bee 7 began to depart from baseline performance at visit 11. Bee 2 began to depart from baseline performance at visit 15.
(TIF)

Figure S3 **0-10-10-0 Individual Inter-Visit-Interval.** All ten experimental 0-10-10-0 individual cumulative curves are presented. All ten experimental 0-10-10-0 bees’ baselines are very similar. Four bees finished all 24 trials with six dropping out. Bee 6 dropped out at visit 8; Bee 10 at visit 9; Bee 8 at visit 13; Bee 9 at visit 15, and Bees 2 and 7 at visit 18. Bee 1 was the only individual not affected by the delay intervals. The remaining three bees (Bees 3, 4, and 5) that were affected by the delay intervals and did finish the 24 trials all saw an immediate near-return to baseline once the delay intervals were removed. Bee 6 began to depart from baseline performance at visit 7. Bees 2, 3, 5, and 10 began to depart from baseline performance at visit 9. Bee 7 began to depart from baseline performance at visit 11. Bee 4 began to depart from baseline performance at visit 16.
(TIF)

Figure S4 **0-5-10-0 Individual Inter-Visit-Interval.** All ten experimental 0-5-10-0 individual cumulative curves are presented. All ten experimental 0-5-10-0 bees’ baselines are very similar. Six bees finished all 24 trials with four dropping out. Bee 4 dropped out at visit 14; Bees 6 and 7 at visit 15; and Bee 18 at visit 18. Bees 2 and 3 were the only individuals not affected by the delay intervals. The remaining four bees (Bees 5, 8, 9, and 10) that were affected by the delay intervals and did finish the 24 trials all saw an immediate near-return to baseline once the delay intervals were removed. Bee 4 began to depart from baseline performance at visit 10. Bees 6 and 7 began to depart from baseline performance at visit 14. Bees 1, 8, 9 and 10 began to depart from baseline performance at visit 15. Bee 5 began to depart from baseline performance at visit 18.
(TIF)

Figure S5 **0-10-5-0 Individual Inter-Visit-Interval.** All ten experimental 0-10-5-0 individual cumulative curves are presented. All ten experimental 0-10-5-0 bees’ baselines are very similar. Five bees finished all 24 trials with five dropping out. Bee 7 dropped out at visit 9; Bees 8 and 10 at visit 12; Bee 1 at visit 16; and Bee 9 at visit 18. Bee 5 was the only individual not affected by the delay intervals. The remaining four bees (Bees 2, 3, 4, and 6) that were affected by the delay intervals and did finish the 24 trials all saw an immediate near-return to baseline once the delay intervals were removed. Bees 7 and 8 began to depart from baseline performance at visit 8. Bees 1, 3, 6, and 9 began to depart from baseline performance at visit 10. Bees 1, 8, 9 and 10 began to depart from baseline performance at visit 15. Bees 2 and 4 began to depart from baseline performance at visit 17.
(TIF)

Figure S6 **0-0-0-0 Temperature and Inter-Visit-Interval.** A scatter-plot of every control 0-0-0-0 bee’s longest inter-visit-interval and the temperature inside the operant chamber of the apparatus during an individual bee’s longest inter-visit-interval is presented. The highest temperature corresponds with the longest inter-visit-interval, while the second longest inter-visit-interval corresponds with the lowest temperature.
(TIF)

Figure S7 **0-5-5-0 Temperature and Individual Inter-Visit-Interval.** A scatter-plot of every experimental 0-5-5-0 bee’s longest inter-visit-interval and the temperature inside the operant chamber of the apparatus during an individual bee’s longest inter-visit-interval is presented.
(TIF)

Figure S8 **0-10-10-0 Temperature and Individual Inter-Visit-Interval.** A scatter-plot of every experimental 0-10-10-0 bee’s longest inter-visit-interval and the temperature inside the operant chamber of the apparatus during an individual bee’s longest inter-visit-interval is presented.
(TIF)

Figure S9 **0-5-10-0 Temperature and Individual Inter-Visit-Interval.** A scatter-plot of nine experimental 0-5-10-0 bee’s longest inter-visit-interval and the temperature inside the operant chamber of the apparatus during an individual bee’s longest inter-visit-interval is presented. The data logger did not record temperature for one 0-5-10-0 bee.
(TIF)

Figure S10 **0-10-5-0 Temperature and Individual Inter-Visit-Interval.** A scatter-plot of nine experimental 0-10-5-0 bee’s longest inter-visit-interval and the temperature inside the operant chamber of the apparatus during an individual bee’s longest inter-visit-interval is presented. The data logger did not record temperature for one 0-10-5-0 bee. Save one data point, a clear trend is easily observed.
(TIF)

Figure S11 **0-0-0-0 Individual Inter-Response-Times.** Individual control 0-0-0-0 bees’ averaged inter-response-times for each visit are presented. Aside from the minor and unsystematic fluctuations of Bees 6 and 9, a stable IRT across the 24 sessions can be observed.
(TIF)

Figure S12 **0-5-5-0 Individual Inter-Response-Times.** Individual experimental 0-5-5-0 bees’ averaged inter-response-times for each visit are presented. Aside from minor and unsystematic fluctuations, a clear decrease in group-average IRT across the 24 sessions can be observed; though no individual decreases are observed. Interestingly, prior to dropping out, Bees 5 and 9 had a vast increase in average inter-response-time. Bee 4¢s session was accidently prematurely terminated at visit 23; thus accounting for the sudden rise in her final IRT.
(TIF)

Figure S13 **0-10-10-0 Individual Inter-Response-Times.** Individual experimental 0-10-10-0 bees’ averaged inter-response-times for each visit are presented. A clear decrease in IRT across the 24 sessions is not observed.
(TIF)

Figure S14 **0-5-10-0 Individual Inter-Response-Times.** Individual experimental 0-5-10-0 bees’ averaged inter-response-times for each visit are presented. Aside from minor and unsystematic fluctuations, a clear decrease in IRT across the 24 sessions can be observed.
(TIF)

Figure S15 **0-10-5-0 Individual Inter-Response-Times.** Individual experimental 0-10-5-0 bees’ averaged inter-response-times for each visit are presented. A clear decrease in IRT across the 24 sessions is not observed.
(TIF)

Figure S16 **0-0-0-0 Individual Response Rate.** Individual control 0-0-0-0 bees’ response rates for each visit are presented. Aside from minor and unsystematic fluctuations, a clear increase in response rate across the 24 sessions can be observed.
(TIF)

Figure S17 **0-5-5-0 Individual Response Rate.** Individual experimental 0-5-5-0 bees’ response rates for each visit are presented. Aside from minor and unsystematic fluctuations, an increase in response rate across the 24 sessions can be interpreted. Interestingly, Bees 5 and 9¢s response rate dips prior to dropping out.
(TIF)

Figure S18 **0-10-10-0 Individual Response Rate.** Individual experimental 0-10-10-0 bees’ response rates for each visit are presented. The 0-10-10-0 group average presented in Figure 4 is easily explained by Bee 6¢s “outlier” response rate data.
(TIF)

Figure S19 **0-5-10-0 Individual Response Rate.** Individual experimental 0-5-10-0 bees’ response rates for each visit are presented. Aside from minor and unsystematic fluctuations, a clear increase in response rate across the 24 sessions can be observed with the removal of the delays. Additionally, there appears to be less variability in response rate during the final condition. Interestingly, Bees 6 and 7 display a dip in response rate prior to dropping out.
(TIF)

Figure S20 **0-10-5-0 Individual Response Rate.** Individual experimental 0-10-5-0 bees’ response rates for each visit are presented. Aside from major and unsystematic fluctuations, a clear increase in response rate across the 24 sessions can be observed with the removal of the delays. Interestingly, Bee 9 displays a dip in response rate prior to dropping out.
(TIF)

## References

[1] Menzel R (2001) Searching for the memory trace in a mini-brain, the honeybee. Learn Mem 8: 53–62. 11274250 10.1101/lm.38801

[2] Giurfa M (2003) Cognitive neuroethology: Dissecting non-elemental learning in a honey bee brain. Curr Opin Neurobiol 13: 726–735. 14662375 10.1016/j.conb.2003.10.015

[3] Giurfa M (2007) Behavioral and neural analysis of associative learning in the honeybee: a taste from the magic well. J Comp Physiol A 193: 801–824. 10.1007/s00359-007-0235-9 17639413

[4] Sanderson CE, Orozco BS, Hill PSM, Wells H (2006) Honeybee (*Apis mellifera* ligustica) response to differences in handling time, rewards, and ﬂower colours. Ethology 112: 937–946.

[5] Menzel R (1999) Memory dynamics of the honeybee. J Comp Physiol A 185: 323–340.

[6] Giray T, Robinson GE (1996) Common endocrine and genetic mechanisms of behavioral development in male and worker honey bees and the evolution of division of labor. Proc Natl Acad Sci U S A 93: 11718–11772. 8876203 10.1073/pnas.93.21.11718 PMC38124

[7] Sokolowski MB, Abramson CI, Craig DPA (2012) Ethanol self-administration in free-flying honeybees (*Apis mellifera* L.) in an operant conditional protocol. Alcohol Clin Exp Res (in press). 10.1111/j.1530-0277.2012.01770.x 22471300

[8] Abramson CI, Wells H, Bozic J (2007) A social insect model for the study of ethanol induced behavior: The honey bee. In: Yoshida r, editor, Trends in Alcohol Abuse and Alcoholism Research. Hauppauge NY: Nova Science Publishers. 197–218.

[9] Galizia CG, Eisenhardt D, Giurfa M (2011) Honeybee neurobiology and behavior: A tribute or Randolf Menzel. New York NY: Springer.

[10] Abbott KR, Dukas R (2009) Honeybees consider flower danger in their waggle dance. Anim Behav 78: 633–635.

[11] Vergoz V, Rousel E, Sandoz JC, Giurfa M (2007) Aversive learning in honeybees revealed by the olfactory conditioning of the sting extension reflex. PLoS ONE 2: e288. 17372627 10.1371/journal.pone.0000288 PMC1810431

[12] Dyer FC, Gill M, Sharbowski J (2002) Motivation and vector navigation in honey bees. Naturwissenschaften 89: 262–264. 12146791 10.1007/s00114-002-0311-5

[13] Davis AD (1954) A test of one aspect of contiguity theory. J Exp Psychol 48: 275–277. 13211938 10.1037/h0060738

[14] Fehrer E (1956) Effects of amount of reinforcement and of pre- and postreinforcment delays on learning and extinction. J Exp Psychol 52: 167–176. 13357699 10.1037/h0045052

[15] Cogan DC (1966) Post-reinforcement delay in extinction: A failure to replicate. Psychonomic Science 6: 343–344.

[16] Bowen J (1966) Effect of post-reward confinement on choice behavior. Psychonomic Science 6: 131.

[17] Mikulka PJ, Vogel JR, Spear NE (1967) Postconsummatory delay and goal box confinement. Psychonomic Science 9: 381–382.

[18] Williams RL (1967) Response strength as a function of pre- and post- reward delay and physical confinement. J Exp Psychol 74: 420–424. 6052622 10.1037/h0024727

[19] McCain G, Bowen J (1967) Pre- and post-reinforcement delay with a small number of acquisition trails. Psychonomic Science 7: 121–122.

[20] Rosen AJ, Tessell RE (1968) Incentive shift and post-reinforcement delay in the runway. Psychol Rep 23: 107–110. 5685376 10.2466/pr0.1968.23.1.107

[21] Capaldi EJ, Godbout RC, Ksir C (1968) A comparison of two delay of reward procedures, pre-reinforcement delay vs post-reinforcement delay. Psychonomic Science 13: 279–280.

[22] Posey TB, Cogan DC (1970) Postreinforcement delay training effect on runway speed patterning. Psychonomic Science 21: 45–47.

[23] Rabinowitz FM, Paynter ML (1969) Postreinforcement interval, intertrail interval, and the delay-retention effect under distraction conditions. J Exp Psychol 81: 177–184.

[24] Hart AG, Ratnieks FLW (2001) Why do honey-bee (*Apis mellifera*) foragers transfer nectar to several receivers? Information improvement through multiple sampling in a biological system. Behav Ecol Sociobiol 49: 244–250.

[25] Farina WM (1995) Food-exchange by foragers in the hive – a means of communication among honey bees? Behav Ecol Sociobiol 38: 59–64.

[26] Grice JW (2011) Observation oriented modeling: Analysis of cause in the behavioral sciences. San Diego CA: Academic Press.

[27] Grice JW, Barrett PT, Schlimgen LA, Abramson CI (2012) Toward a brighter future for psychology as an observation oriented science. Behavioral Sciences 2: 1–22. 25379212 10.3390/bs2010001 PMC4217578

[28] Heinrich B (1979) Keeping a cool head: Honeybee thermoregulation. Science 21: 1269–1271. 10.1126/science.205.4412.1269 17750151

[29] Chung SH (1965) Effects of delayed reinforcement in a concurrent situation. J Appl Behav Anal 8: 439–444. 10.1901/jeab.1965.8-439 PMC1338130 5851405

[30] Chung SH, Herrnstein RJ (1967) Choice and delay of reinforcement. J Appl Behav Anal 8: 67–74. 10.1901/jeab.1967.10-67 PMC1338319 16811307

[31] Sizemore OJ, Lattal KA (1978) Unsignalled delay of reinforcement in variable-interval schedules. J Appl Behav Anal 30: 169–175. 10.1901/jeab.1978.30-169 PMC1332712 16812096

[32] Abramson CI (1990) Invertebrate Learning: A Laboratory Manual and Source Book. Washington DC: American Psychological Association.

[33] Nunez JA, Giurfa M (1996) Motivation and regulation of honey bee foraging. Bee World 77: 182–196.

[34] Moffatt L (2000) Changes in the metabolic rate of the foraging honeybee: Effect of the carried weight or of the reward rate? J Comp Physiol A 186: 299–306. 10757245 10.1007/s003590050430

[35] Chen YL, Hung YS, Yang EC (2008) Biogenic amine levels change in the brains of stressed honeybees. Arch Insect Biochem Physiol 68: 241–250. 18618764 10.1002/arch.20259

[36] Visscher PK, Seeley TD (1982) Foraging strategy of honeybee colonies in a temperate deciduous forest. Ecology 63: 1790–1801.

[37] Bentley B, Elias T, editors (1983) The biology of nectaries. New York NY: Columbia University Press.

[38] Graham JM, editor (1992) The Hive and the Honey Bee. Hamilton IL: Dadan & Sons.

[39] Wells H, Wells PH, Smith DM (1981) Honey bee response to reward size and color in an artificial flower patch. J Apic Res 20: 172–179.

[40] Sanderson CE, Orozco BS, Hill PSM, Wells H (2006) Honey bee (*Apis mellifera* ligustica) response to differences in handling time, rewards and colors. Ethology 112: 937–946.

[41] Wells H, Wells PH (1986) Optimal diet, minimal uncertainty and individual constancy in the foraging of honey bees. J Anim Ecol 55: 881–891.

[42] Wells H, Hill PS, Wells PH (1992) Nectivore foraging ecology: Rewards differing in sugar type. Ecol Entomol 17: 280–288.

[43] Bronstein PM (1981) Social reinforcement in *Betta splendens*: A reconsideration. J Comp Physiol Psychol 95: 943–950.

[44] Bronstein PM (1983) Agonistic sequences nad the assessment of opponents in male *Betta splendens* . Am J Psychol 96: 163–177.

[45] Bronstein PM (2006) Toxiphobia, “social reinforcement,” comparative psychology, and Patrick J. Capretta. Ann N Y Acad Sci 443: 158–170. 10.1111/j.1749-6632.1985.tb27071.x 3860069

[46] Robinson GE, Fernald RD, Clayton DF (2008) Genes and Social Behavior. Science 322: 896–900. 18988841 10.1126/science.1159277 PMC3052688

[47] De Marco RJ (2006) How bees tune their dancing according to their colony’s nectar influx: Re-examining the role of the food-receivers’ ‘eagerness’. J Exp Biol 209: 421–432. 16424092 10.1242/jeb.02025

[48] Doolittle GM (1907) Where do the field-bees deposit their loads? Am Bee J 42: 653–654.

[49] Ratnieks FLW, Anderson A (1999) Task partitioning in insect societies. II. Use of queuing delay information in social insects. Am Nat 154: 536–548. 10561126 10.1086/303256

[50] Anderson C, Ratnieks FLW (1999) Worker allocation in insect societies: Coordination of nectar foragers and nectar receivers in honey bee (*Apis mellifera*) colonies. Behav Ecol Sociobiol 46: 73–81.

[51] Wainselboim AJ, Roces F, Farina WM (2003) Assessment of food source profitability in honeybees (*Apis mellifera*): how does disturbance of foraging activity affect trophallactic behaviour? J Comp Physiol 189: 39–45. 12548428 10.1007/s00359-002-0373-z

[52] Cakmak I, Song DS, Mixson TA, Serrano E, Clement ML, et al (2010) Foraging response of honeybee subspecies to flower color choices and reward consistency. Insect Behavior 23: 100–116.

[53] Sokolowski MB, Abramson CI (2010) From foraging to operant conditioning: A new computer-controlled Skinner box to study free-flying nectar gathering behavior in bees. J Neurosci Methods 188: 235–242. 20171985 10.1016/j.jneumeth.2010.02.013

