# Supplementary figures and images for: Social Reinforcement Delays in Free-Flying Honey Bees (Apis mellifera L.)
Source: PLoS One. 2012 Oct 4;7(10):e46729. doi: 10.1371/journal.pone.0046729 (PMC3464271; doi:10.1371/journal.pone.0046729)

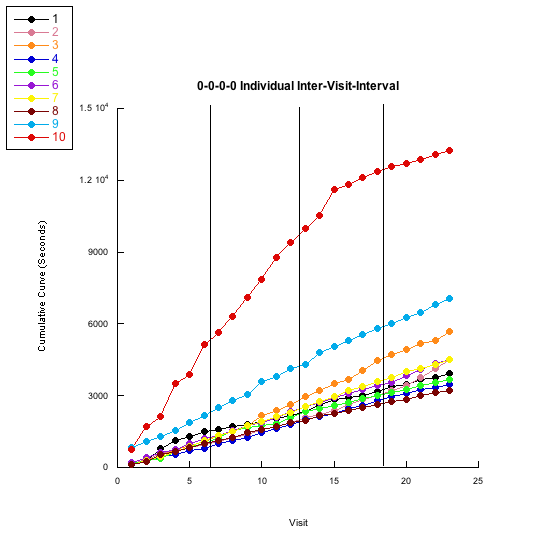

Supplement: Figure S1 — 0-0-0-0 Individual Inter-Visit-Interval. All ten control 0-0-0-0 individual cumulative curves are presented. Bee 10 is the only subject not following pattern mode for the control 0-0-0-0 group; indeed a learning curve could be interpreted. Bee 10 begins resembling the other 9 control 0-0-0-0 bees by visit 15. (TIF) [file pone.0046729.s001.tif]

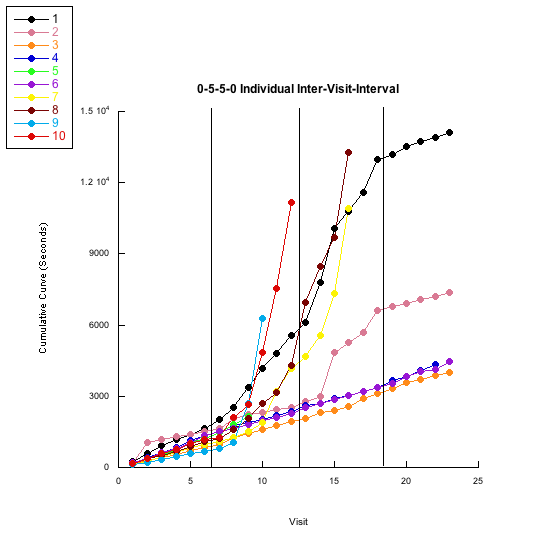

Supplement: Figure S2 — 0-5-5-0 Individual Inter-Visit-Interval. All ten experimental 0-5-5-0 individual cumulative curves are presented. All ten experimental 0-5-5-0 bees’ baselines are very similar. Five bees finished all 24 trials with five dropping out. Bees 5 and 9 dropped out at visit 9; Bee 10 at visit 12; and Bee 7 and 8 at visit 16. Bee 5 is the only bee that did not return to the nearby bee-feeder instead of the apparatus; hence a jump of 3600 s is not observed for this cumulative curve. Bees 3, 4, and 6 were not affected by the delay intervals. The remaining two bees that were affected by the delay intervals and did finish the 24 trials both saw an immediate near-return to baseline once the delay intervals were removed. Bees 1, 8, 9, and 10 began to depart from baseline performance as early as visit 8. Bee 7 began to depart from baseline performance at visit 11. Bee 2 began to depart from baseline performance at visit 15. (TIF) [file pone.0046729.s002.tif]

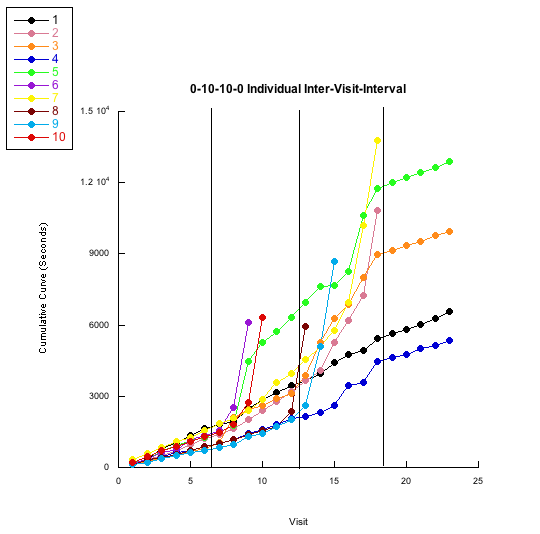

Supplement: Figure S3 — 0-10-10-0 Individual Inter-Visit-Interval. All ten experimental 0-10-10-0 individual cumulative curves are presented. All ten experimental 0-10-10-0 bees’ baselines are very similar. Four bees finished all 24 trials with six dropping out. Bee 6 dropped out at visit 8; Bee 10 at visit 9; Bee 8 at visit 13; Bee 9 at visit 15, and Bees 2 and 7 at visit 18. Bee 1 was the only individual not affected by the delay intervals. The remaining three bees (Bees 3, 4, and 5) that were affected by the delay intervals and did finish the 24 trials all saw an immediate near-return to baseline once the delay intervals were removed. Bee 6 began to depart from baseline performance at visit 7. Bees 2, 3, 5, and 10 began to depart from baseline performance at visit 9. Bee 7 began to depart from baseline performance at visit 11. Bee 4 began to depart from baseline performance at visit 16. (TIF) [file pone.0046729.s003.tif]

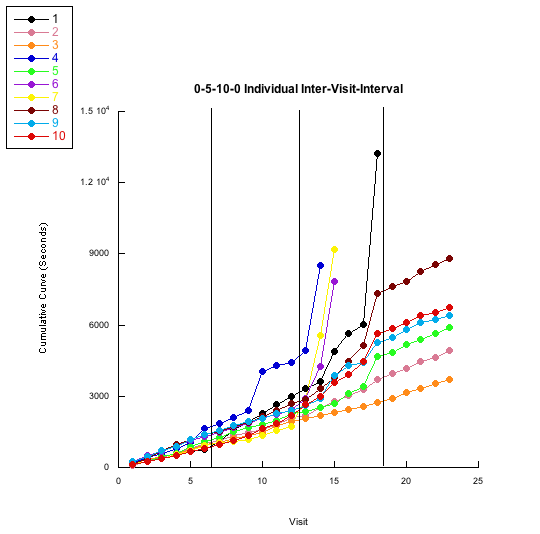

Supplement: Figure S4 — 0-5-10-0 Individual Inter-Visit-Interval. All ten experimental 0-5-10-0 individual cumulative curves are presented. All ten experimental 0-5-10-0 bees’ baselines are very similar. Six bees finished all 24 trials with four dropping out. Bee 4 dropped out at visit 14; Bees 6 and 7 at visit 15; and Bee 18 at visit 18. Bees 2 and 3 were the only individuals not affected by the delay intervals. The remaining four bees (Bees 5, 8, 9, and 10) that were affected by the delay intervals and did finish the 24 trials all saw an immediate near-return to baseline once the delay intervals were removed. Bee 4 began to depart from baseline performance at visit 10. Bees 6 and 7 began to depart from baseline performance at visit 14. Bees 1, 8, 9 and 10 began to depart from baseline performance at visit 15. Bee 5 began to depart from baseline performance at visit 18. (TIF) [file pone.0046729.s004.tif]

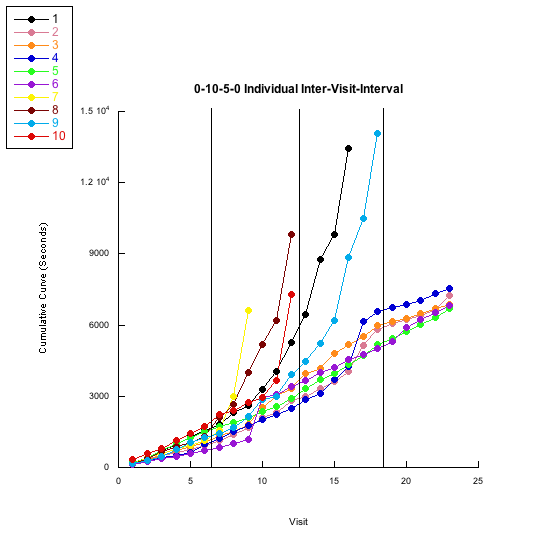

Supplement: Figure S5 — 0-10-5-0 Individual Inter-Visit-Interval. All ten experimental 0-10-5-0 individual cumulative curves are presented. All ten experimental 0-10-5-0 bees’ baselines are very similar. Five bees finished all 24 trials with five dropping out. Bee 7 dropped out at visit 9; Bees 8 and 10 at visit 12; Bee 1 at visit 16; and Bee 9 at visit 18. Bee 5 was the only individual not affected by the delay intervals. The remaining four bees (Bees 2, 3, 4, and 6) that were affected by the delay intervals and did finish the 24 trials all saw an immediate near-return to baseline once the delay intervals were removed. Bees 7 and 8 began to depart from baseline performance at visit 8. Bees 1, 3, 6, and 9 began to depart from baseline performance at visit 10. Bees 1, 8, 9 and 10 began to depart from baseline performance at visit 15. Bees 2 and 4 began to depart from baseline performance at visit 17. (TIF) [file pone.0046729.s005.tif]

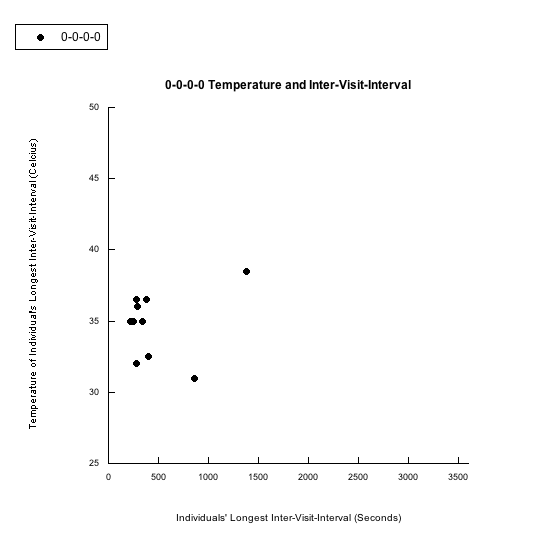

Supplement: Figure S6 — 0-0-0-0 Temperature and Inter-Visit-Interval. A scatter-plot of every control 0-0-0-0 bee’s longest inter-visit-interval and the temperature inside the operant chamber of the apparatus during an individual bee’s longest inter-visit-interval is presented. The highest temperature corresponds with the longest inter-visit-interval, while the second longest inter-visit-interval corresponds with the lowest temperature. (TIF) [file pone.0046729.s006.tif]

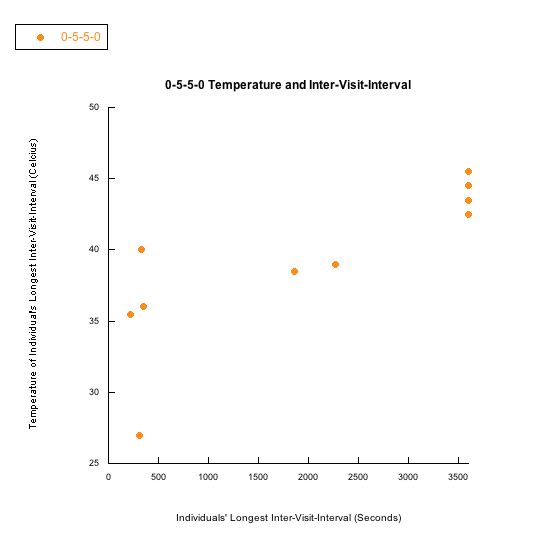

Supplement: Figure S7 — 0-5-5-0 Temperature and Individual Inter-Visit-Interval. A scatter-plot of every experimental 0-5-5-0 bee’s longest inter-visit-interval and the temperature inside the operant chamber of the apparatus during an individual bee’s longest inter-visit-interval is presented. (TIF) [file pone.0046729.s007.tif]

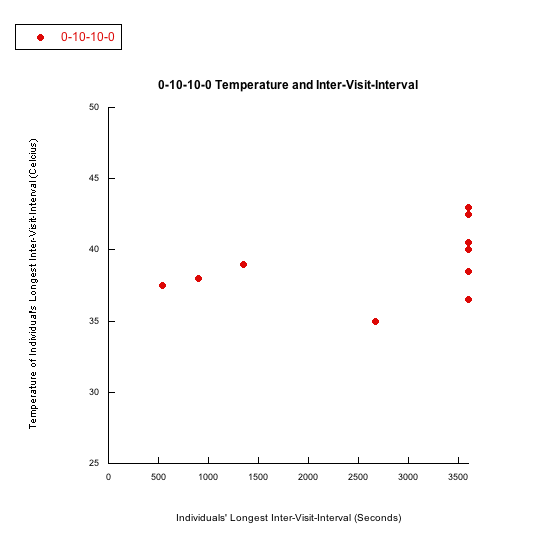

Supplement: Figure S8 — 0-10-10-0 Temperature and Individual Inter-Visit-Interval. A scatter-plot of every experimental 0-10-10-0 bee’s longest inter-visit-interval and the temperature inside the operant chamber of the apparatus during an individual bee’s longest inter-visit-interval is presented. (TIF) [file pone.0046729.s008.tif]

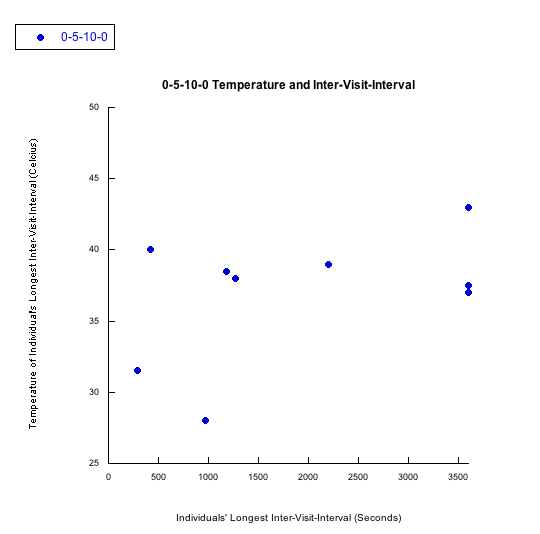

Supplement: Figure S9 — 0-5-10-0 Temperature and Individual Inter-Visit-Interval. A scatter-plot of nine experimental 0-5-10-0 bee’s longest inter-visit-interval and the temperature inside the operant chamber of the apparatus during an individual bee’s longest inter-visit-interval is presented. The data logger did not record temperature for one 0-5-10-0 bee. (TIF) [file pone.0046729.s009.tif]

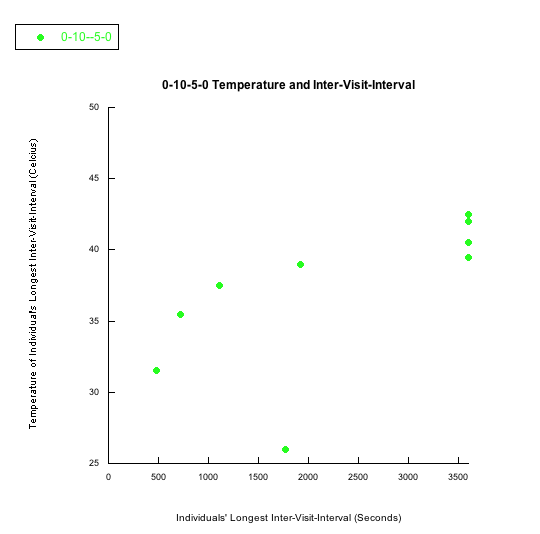

Supplement: Figure S10 — 0-10-5-0 Temperature and Individual Inter-Visit-Interval. A scatter-plot of nine experimental 0-10-5-0 bee’s longest inter-visit-interval and the temperature inside the operant chamber of the apparatus during an individual bee’s longest inter-visit-interval is presented. The data logger did not record temperature for one 0-10-5-0 bee. Save one data point, a clear trend is easily observed. (TIF) [file pone.0046729.s010.tif]

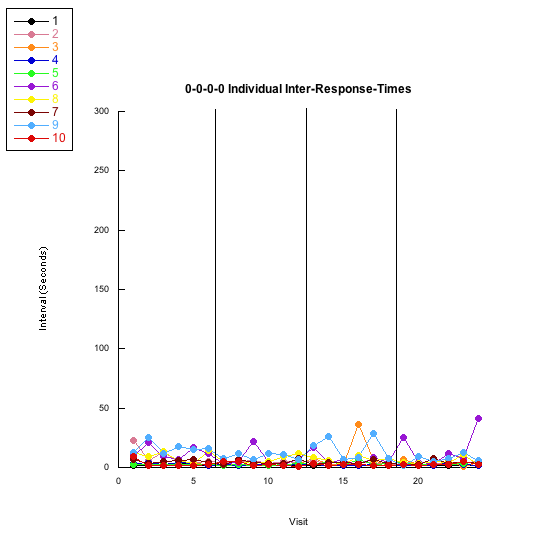

Supplement: Figure S11 — 0-0-0-0 Individual Inter-Response-Times. Individual control 0-0-0-0 bees’ averaged inter-response-times for each visit are presented. Aside from the minor and unsystematic fluctuations of Bees 6 and 9, a stable IRT across the 24 sessions can be observed. (TIF) [file pone.0046729.s011.tif]

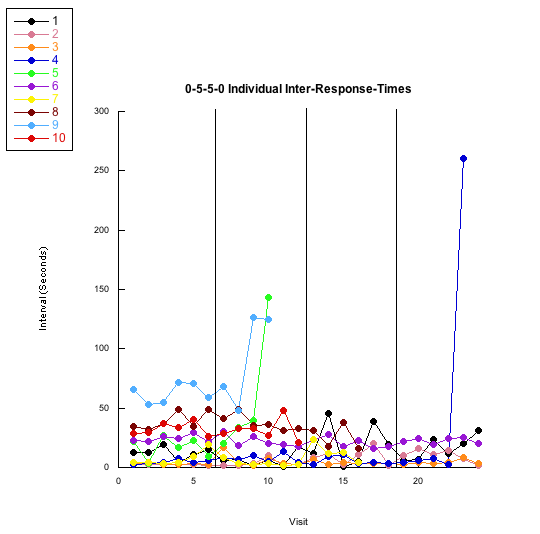

Supplement: Figure S12 — 0-5-5-0 Individual Inter-Response-Times. Individual experimental 0-5-5-0 bees’ averaged inter-response-times for each visit are presented. Aside from minor and unsystematic fluctuations, a clear decrease in group-average IRT across the 24 sessions can be observed; though no individual decreases are observed. Interestingly, prior to dropping out, Bees 5 and 9 had a vast increase in average inter-response-time. Bee 4¢s session was accidently prematurely terminated at visit 23; thus accounting for the sudden rise in her final IRT. (TIF) [file pone.0046729.s012.tif]

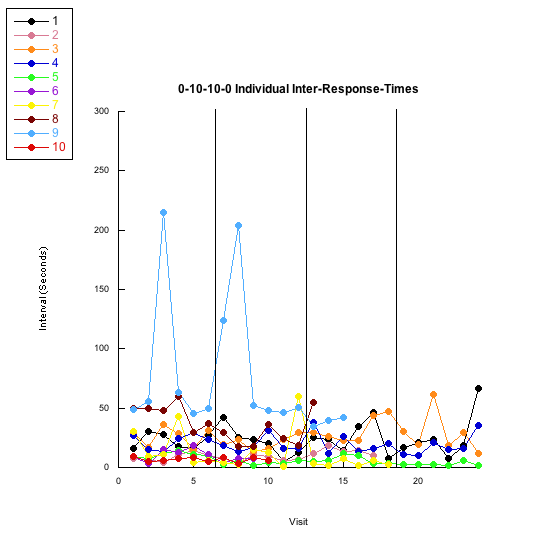

Supplement: Figure S13 — 0-10-10-0 Individual Inter-Response-Times. Individual experimental 0-10-10-0 bees’ averaged inter-response-times for each visit are presented. A clear decrease in IRT across the 24 sessions is not observed. (TIF) [file pone.0046729.s013.tif]

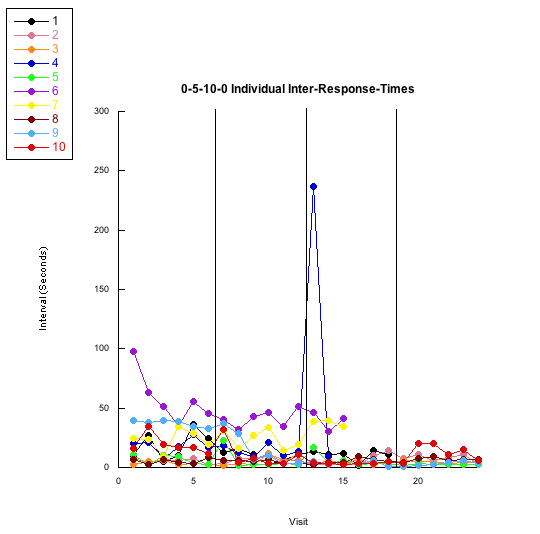

Supplement: Figure S14 — 0-5-10-0 Individual Inter-Response-Times. Individual experimental 0-5-10-0 bees’ averaged inter-response-times for each visit are presented. Aside from minor and unsystematic fluctuations, a clear decrease in IRT across the 24 sessions can be observed. (TIF) [file pone.0046729.s014.tif]

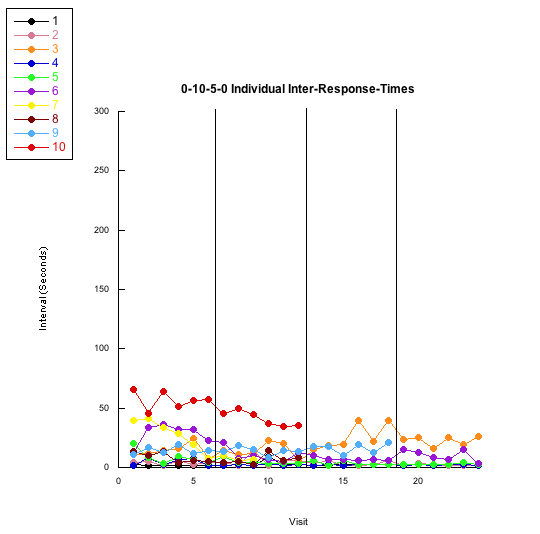

Supplement: Figure S15 — 0-10-5-0 Individual Inter-Response-Times. Individual experimental 0-10-5-0 bees’ averaged inter-response-times for each visit are presented. A clear decrease in IRT across the 24 sessions is not observed. (TIF) [file pone.0046729.s015.tif]

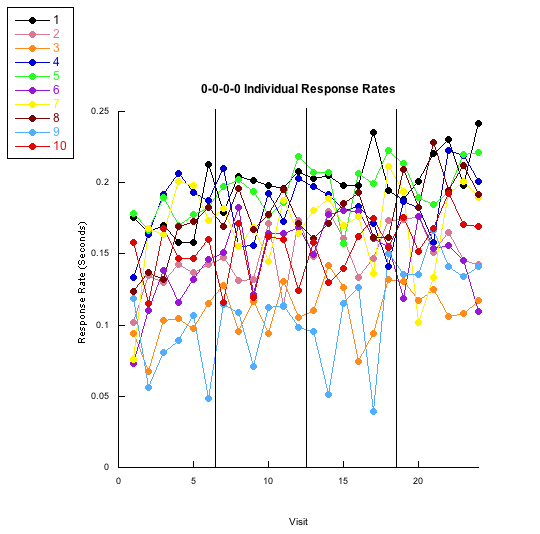

Supplement: Figure S16 — 0-0-0-0 Individual Response Rate. Individual control 0-0-0-0 bees’ response rates for each visit are presented. Aside from minor and unsystematic fluctuations, a clear increase in response rate across the 24 sessions can be observed. (TIF) [file pone.0046729.s016.tif]

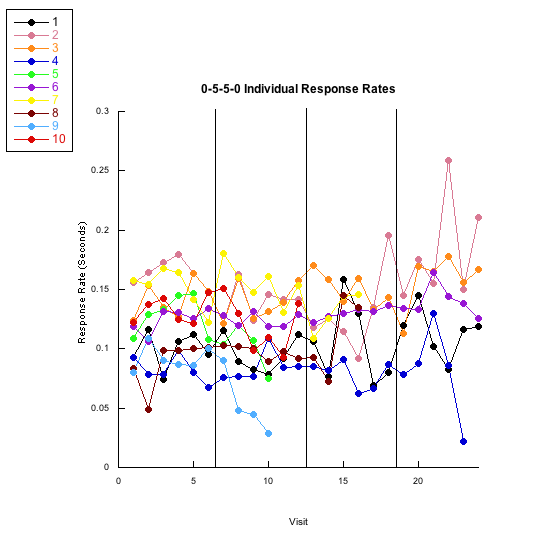

Supplement: Figure S17 — 0-5-5-0 Individual Response Rate. Individual experimental 0-5-5-0 bees’ response rates for each visit are presented. Aside from minor and unsystematic fluctuations, an increase in response rate across the 24 sessions can be interpreted. Interestingly, Bees 5 and 9¢s response rate dips prior to dropping out. (TIF) [file pone.0046729.s017.tif]

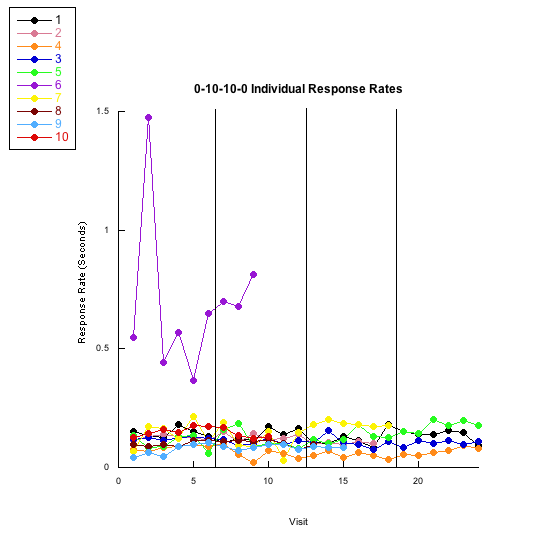

Supplement: Figure S18 — 0-10-10-0 Individual Response Rate. Individual experimental 0-10-10-0 bees’ response rates for each visit are presented. The 0-10-10-0 group average presented in Figure 4 is easily explained by Bee 6¢s “outlier” response rate data. (TIF) [file pone.0046729.s018.tif]

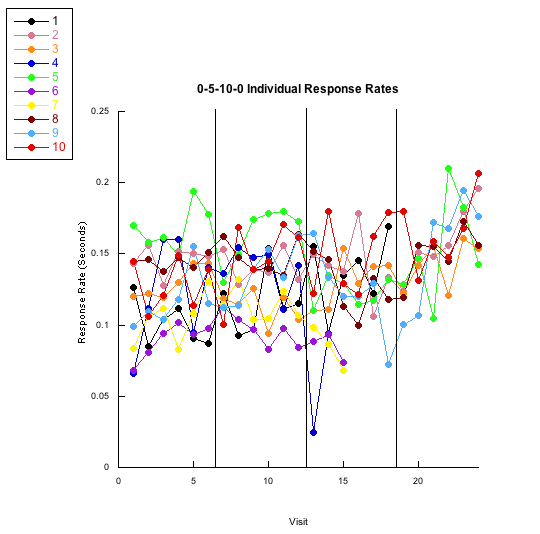

Supplement: Figure S19 — 0-5-10-0 Individual Response Rate. Individual experimental 0-5-10-0 bees’ response rates for each visit are presented. Aside from minor and unsystematic fluctuations, a clear increase in response rate across the 24 sessions can be observed with the removal of the delays. Additionally, there appears to be less variability in response rate during the final condition. Interestingly, Bees 6 and 7 display a dip in response rate prior to dropping out. (TIF) [file pone.0046729.s019.tif]

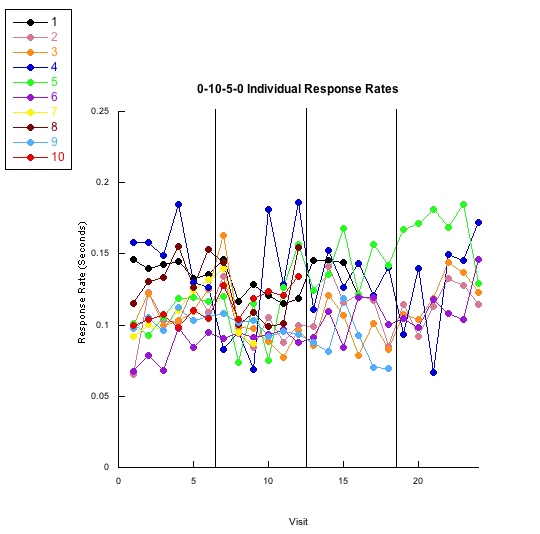

Supplement: Figure S20 — 0-10-5-0 Individual Response Rate. Individual experimental 0-10-5-0 bees’ response rates for each visit are presented. Aside from major and unsystematic fluctuations, a clear increase in response rate across the 24 sessions can be observed with the removal of the delays. Interestingly, Bee 9 displays a dip in response rate prior to dropping out. (TIF) [file pone.0046729.s020.tif]
